# *Plasmodium falciparum* gametocyte carriage in longitudinally monitored incident infections is associated with duration of infection and human host factors

**DOI:** 10.1038/s41598-023-33657-3

**Published:** 2023-05-01

**Authors:** Chiara Andolina, Jordache Ramjith, John Rek, Kjerstin Lanke, Joseph Okoth, Lynn Grignard, Emmanuel Arinaitwe, Jessica Briggs, Jeffrey Bailey, Ozkan Aydemir, Moses R. Kamya, Bryan Greenhouse, Grant Dorsey, Sarah G. Staedke, Chris Drakeley, Marianne Jonker, Teun Bousema

**Affiliations:** 1 Department of Medical Microbiology, Radboud University Nijmegen Medical Centre, Nijmegen, The Netherlands; 2 Department for Health Evidence, Radboud University Medical Centre, Nijmegen, The Netherlands; 3 Infectious Diseases Research Collaboration, Kampala, Uganda; 4 Department of Infection Biology, London School of Hygiene and Tropical Medicine, London, UK; 5 Department of Medicine, San Francisco General Hospital, University of California, San Francisco, USA; 6 Department of Pathology and Laboratory Medicine, Brown University, Providence, RI USA; 7 Program in Molecular Medicine, University of Massachusetts Medical School, Worcester, MA USA; 8 Department of Medicine, Makerere University College of Health Sciences, Kampala, Uganda; 9 Department of Clinical Research, London School of Hygiene and Tropical Medicine, London, UK

**Keywords:** Computational biology and bioinformatics, Microbiology

## Abstract

Malaria transmission depends on the presence of *Plasmodium* gametocytes that are the only parasite life stage that can infect mosquitoes. Gametocyte production varies between infections and over the course of infections. Infection duration is highly important for gametocyte production but poorly quantified. Between 2017 and 2019 an all-age cohort of individuals from Tororo, eastern Uganda was followed by continuous passive and routine assessments. We longitudinally monitored 104 incident infections from 98 individuals who were sampled once every 28 days and on any day of symptoms. Among infections that lasted ≥ 3 months, gametocyte appearance was near-universal with 96% of infections having detectable gametocytes prior to clearance. However, most infections were of much shorter duration; 55.7% of asymptomatic infections were detected only once. When considering all asymptomatic infections, regardless of their duration, only 36.3% had detectable gametocytes on at least one time-point prior to parasite clearance. Infections in individuals with sickle-cell trait (HbAS) were more likely to have gametocytes detected (Hazard Rate (HR) = 2.68, 95% CI 1.12, 6.38; p = 0.0231) and had gametocytes detected at higher densities (Density Ratio (DR) = 9.19, 95% CI 2.79, 30.23; p = 0.0002) compared to infections in wildtype (HbAA) individuals. Our findings suggest that a large proportion of incident infections is too short in duration and of too low density to contribute to onward transmission.

## Introduction

Despite significant progress in the last decade, *P. falciparum* malaria remains a leading cause of morbidity and mortality in many countries in Sub Saharan Africa. In 2020 there were 241 million malaria cases, 14 million more than in 2019, and the number of deaths increased from 558,000 to 627,000^1^. This was in part attributed to the disruption of malaria prevention and treatment during the COVID-19 pandemic^2^. However, even before the COVID-19 pandemic, global progress against malaria plateaued or even reversed in some endemic regions, related to funding shortages that impacted delivery and access to vector control, early diagnosis and treatment. Control efforts are further threatened by the emergence and spread of drug and insecticide resistance^3,4^; new interventions are therefore urgently needed to sustainably reduce the burden of malaria and move towards elimination. Transmission-blocking interventions are considered highly relevant for elimination purposes and their development and deployment depends on a thorough understanding of human to mosquito transmission.

In human hosts, *P. falciparum* parasites replicate asexually over approximately 48 h, after which merozoites are released into the circulation where they invade new red blood cells. The majority of merozoites continue this asexual cycle, whilst a minority commit to become male or female gametocytes^5^. Gametocytes mature in human host tissues, primarily the bone marrow and spleen^6,7^, until their release into the peripheral circulation approximately 8–12 days after the initial wave of asexual parasites^5^. Mature gametocytes thereby become accessible to mosquitoes taking a blood meal. Once in the mosquito midgut, gametocytes differentiate into gametes that fertilize and, following sporogonic development, ultimately lead to the presence of infective sporozoites in the mosquito salivary glands. The average lifespan of circulating mature gametocytes is 6.5 days^5,8^ but a proportion of gametocytes can persist and be infectious to mosquitoes for several weeks or even months following effective clearance of asexual parasites^8^.

Although gametocytes are formed from their asexual progenitors and gametocyte densities are therefore associated with the preceding asexual parasite biomass, this association is relatively weak because gametocyte production varies between infections^9^ and over the course of infections^10^. The factors that drive gametocyte production are poorly understood. Controlled malaria infections in malaria naïve volunteers suggest that gametocyte production may be highest early after infection, although a comparison of infections during peak and low transmission seasons suggests that gametocyte production relative to the asexual parasite biomass may be higher in the low transmission season^11^ when infections are relatively old^12^. The duration of infection is a critical determinant of gametocyte production and thereby the human infectious reservoir for malaria but is currently poorly characterised^13^. While *P. falciparum* infections can be very short-lived, being detectable for only a few days^14^, most attention has gone to longer-lasting infections that can persist for several months or even years in the absence of treatment^15^. These longer-term infections are considered the most important drivers of malaria transmission in most endemic settings and are often present at low, sometimes submicroscopic, parasite densities. It is generally assumed that all *P. falciparum* infections, including submicroscopic infections, have the potential to produce gametocytes and be infectious to mosquitoes^16^ because of low-level stochastic commitment to gametocyte production^17^. In addition to this stochastic commitment, parasite may increase gametocyte production in response to the stimuli described above. There have been no accurate estimations of the time and magnitude of gametocyte appearance during natural infections; these require repeated sampling and sensitive molecular techniques.

Gametocyte carriage in naturally acquired infections has been associated with younger age, lower parasitemia in clinical cases, lower hemoglobin levels, longer duration of infections (reviewed in^13,18^). The investment in gametocyte production may further differ according to transmission setting^19,20^ and in response to triggers such as antimalarial treatment, inflammation, availability of host cells or immune clearance that prompt parasites to prioritize either within-host survival (i.e. sustain or increase asexual multiplication by reducing gametocyte commitment) or the immediate transmission potential to mosquitoes (e.g. as part of a terminal investment)^21^. Also human genetic factors have been associated with gametocyte production^22,23^. Sickle cell trait (HbAS) is a common host genetic polymorphism in eastern Uganda, with a prevalence around 30%^27,28^. HbAS is associated with protection against severe malaria disease^29,30^ and may also have implication for malaria transmission. A single longitudinal study in an area of intense malaria transmission in Uganda where gametocytes were examined by microscopy found evidence of higher gametocyte carriage among individuals with the HbAS genotype^27^, a finding that was supported by a cross-sectional study from Senegal^24^. However, other studies observed no association with gametocyte carriage but suggested increased transmissibility of gametocytes from HbAS carriers^23,27,31,32^.

We examined gametocyte carriage over the course of infections in relation to host and parasite characteristics using longitudinal data from 104 incident malaria infections in an all-age cohort in eastern Uganda^33^. Sensitive molecular assays were deployed for the quantification of total parasite density and gametocyte density; amplicon deep sequencing was used to genotype infections and distinguish between incident and persistent infections. Human host and infection characteristics were examined in relation to infection duration and gametocyte appearance.

## Results

### The majority of incident infections were asymptomatic and of short duration

Between 2017 and 2019, all 531 residents from 80 randomly selected households were enrolled into a study cohort followed for up to 24 months^33^. We investigated the duration of incident malaria infections among individuals who were parasite-free by varATS qPCR on 3 previous visits. This qPCR is DNA-based and thus detects both asexual parasites and gametocytes. This approach, that excluded all individuals who were already parasite positive at enrolment, resulted in the identification of 104 newly acquired infections among 98 individuals. Sixteen of these incident infections (15.4%) were symptomatic at the time of first detection, had a geometric mean of 19,817 parasites/µL (95% CI 7,260, 54,096), and received first-line treatment for uncomplicated malaria immediately after sampling. The remainder of incident infections (84.6%) were asymptomatic at first detection and had a geometric mean of 0.87 parasites/µL (95% CI 0.34, 2.21) at the time of initial detection (Table 1); in 13.6% (12/88) of these initially asymptomatic infections, symptoms and parasites detected by microscopy occurred at a later time-point and prompted antimalarial treatment.

**Table 1:** Characteristics of incident infections that presented with symptoms or were initially asymptomatic.

	Incident symptomatic infections	Incident initially asymptomatic infections	p-value
At the moment of infection detection			
Proportion of infections in each class	15.4% (16/104)	84.6% (88/104)	
Hb genotype	1 missing	2 missing	p = 0.1435
AA	73.3% (11/15)	67.4% (58/86)	
SS	20% (3/15)	8.1% (7/86)	
AS	6.6% (1/15)	24.4% (21/86)	
Geometric mean parasite density; parasite/µl (96% CI)	19,817.75 (7260.10, 54,096.12)	0.87 (0.34, 2.21)	p < 0.0001
Gametocyte prevalence	6. 3% (1/16)	16.4% (14/85)^#^	p = 0.5321
During infection			
Geometric mean parasite density; parasite/µl (96% CI)	19,817.75 (7260.10, 54,096.12)	5.53 (1.77, 17.23)	p < 0.0001
Polyclonal infection*	50% (8/16)	33.3% (17/51)	p = 0.2505
Number pf clones, median (range)**	1.5 (1–5)	1 (1–5)	p = 0.1158
Percentage receiving treatment***	100% (16/16)	13.6% (12/88)	p < 0.0001

^#^Three of the 88 initially asymptomatic infections had missing RNA samples at time 0.

*Polyclonal infection was defined as having at least 2 parasite clones based on AMA-1 amplicon sequencing.

**Number of clones was defined as the total number of clones detected by AMA-1 amplicon sequencing. Polyclonal infection and the number of parasite clones were measured across the entire duration of infection; for symptomatic incident infections this equaled the first time-point of detection since treatment was immediately provided. For asymptomatic incident infections, AMA-1 sequencing was unsuccessful for 37 infections.

***Drug treatment was initiated immediately for symptomatic infections, while for incident initially asymptomatic infections treatment occurred in 13.6% of infections during follow-up (i.e. the infection started asymptomatic but developed into a symptomatic episode). For all cells % (n/N) is provided unless stated otherwise.

At enrollment, 95% (505/531) of individuals in the entire cohort were successfully genotyped for sickle cell trait HbAS, including individuals without incident infections. Among these, 25.5% (129/505) had the HbAS genotype and 8.3% (42/505) had the HbSS genotype. These proportions were similar to the prevalence of this haemoglobinopathy among individuals with incident infections, 21.8% HbAS (22/101) and9.9% HbSS (10/101). Among infections in individuals with the HbAS genotype, 4.5% (1/22) were symptomatic upon presentation compared to 30. % (3/10) among HbSS and 15.9% (11/69) among HbAA (Table 1, p = 0.1435).

The duration of infections was estimated based on time to qPCR negativity; if an infection was undetected at a given time-point followed by a qPCR positive visit up to 16 weeks later with the same parasite clone, we considered an infection as persisting. The detection of parasite clones was based on AMA-1 amplicon sequencing. The expected heterozygosity value for AMA-1 was high, H_e_ = 0.949, indicating high genetic variation within the population and thus a low chance of individual infections having the same AMA-1 genotype. Sequencing success was associated with parasite density, with a geometric mean parasite density of 0.03 parasites/µL (95% CI 0.02, 0.05) among samples that failed (n = 37) and 59.00 parasites/µL (95% CI 17.18, 202.69; p < 0.0001) among samples that had successful genotyping results (n = 67) at the initial detection of incident infections. Overall, 37.3% (25/67) of incident infections with genotyping results were polyclonal, i.e. had multiple *P. falciparum* clones detected by AMA-1 amplicon sequencing.

**Figure 1:**
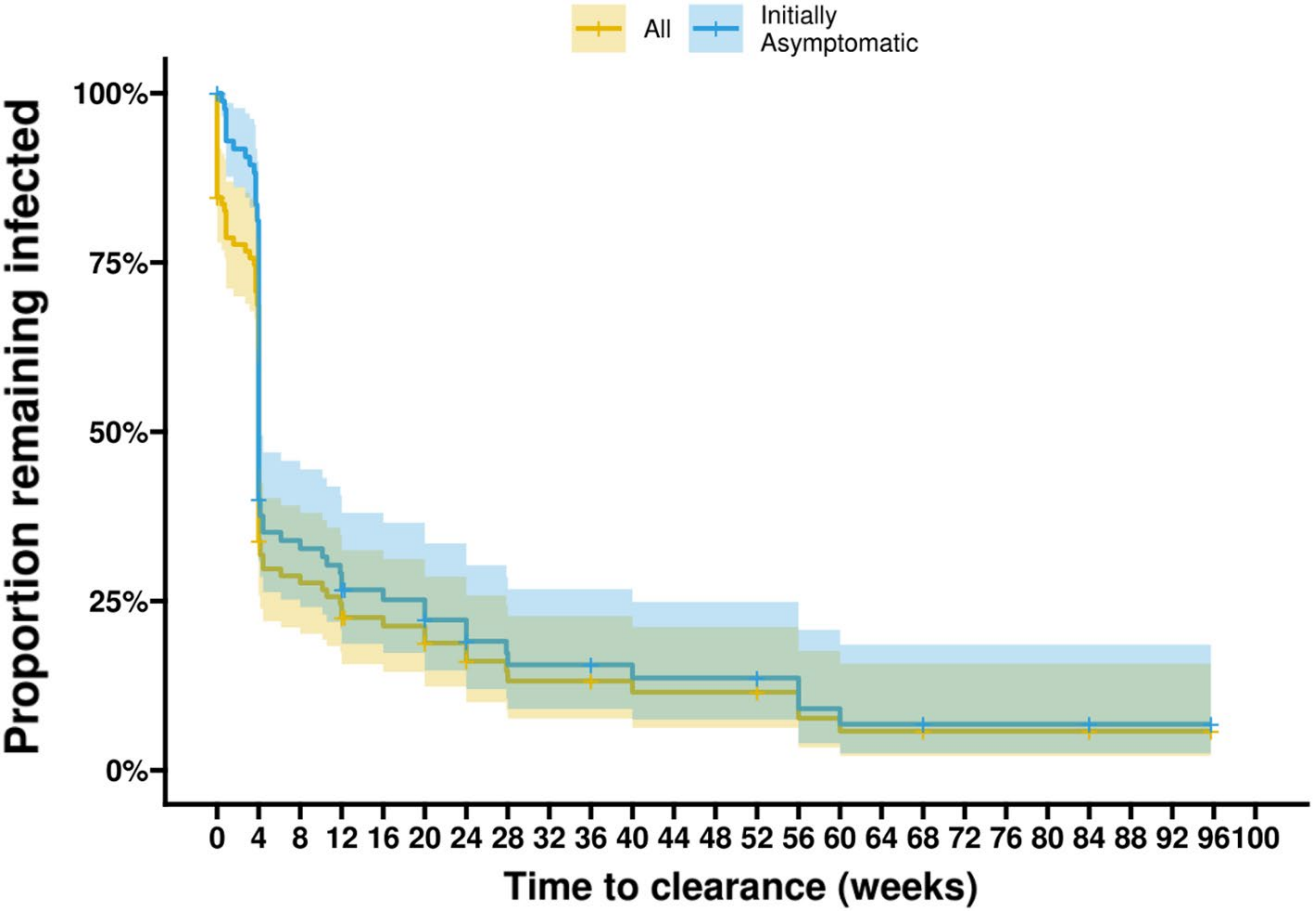
The duration of incident infections. Kaplan–Meier survival curves are shown for all incident infections (yellow) and for all incident infections that were initially asymptomatic (blue). The y-axis shows the probability of remaining infected (i.e. not clearing infection) before a given time (in weeks) reflected on the x-axis. Time to clearance was defined as either the time of onset of clinical symptoms (when the infection was interrupted by treatment) or the first parasite-free visit. From all 104 incident infections, 16 were symptomatic at the initial visit; these were immediately treated and considered cleared; whereas 17% (15/88) of initially asymptomatic infections (and 14.4% (15/104) of all infections) did not clear by the end of follow-up, with the longest follow-up from the initial detection of infection being 96 weeks. This is reflected by the steep drop in persistence for all infections (yellow) at time point 0. We also observed a steep descent in the probability of remaining infected at the 4 weeks visit 71% (35/49) of initially asymptomatic infections that were only detected once(at t = 0) were considered cleared at exactly this time point.

In our cohort, most incident infections were only detected at a single time point (Fig. 1), with a steep decline in survival probabilities after 4 weeks. When focusing only on those infections that were initially asymptomatic (n = 88), and thus did not receive treatment when first detected, 55.7% (49/88) were detected only once. From these 49 infections, 3 were considered right-censored (i.e. they were no subsequent observations, and the remaining 46 were considered cleared at the subsequent visit—approximately 4 weeks (3.6–4.4 weeks). Only 25% (22/88) of infections persisted for ≥ 12 weeks. All 16 initially symptomatic infections received prompt treatment and were thus considered immediately cleared; this was illustrated by the rapid decline in survival probabilities (Fig. 1). The geometric mean parasite density among incident asymptomatic infections that were detected only once was 0.15 parasites/µL (95% CI 0.06, 0.38), which was lower than the initial parasite density of infections that were detected at multiple occasions (2.11 parasites/µL (95% CI 0.62, 3.59; p < 0.0001). Among incident infections that were initially asymptomatic, there was no apparent association between age and infection duration: 65% (13/20), 51.4% (19/37) and 54.8% (17/31) of infections were detected only once for individuals aged < 5, 5–15 and 16 + years old, respectively (Supplemental Fig. S1). Similarly, HbAS genotype did not have a notable effect on infection duration (Supplemental Fig. S1).

### Gametocytes are detected among most long-duration infections

**Figure 2:**
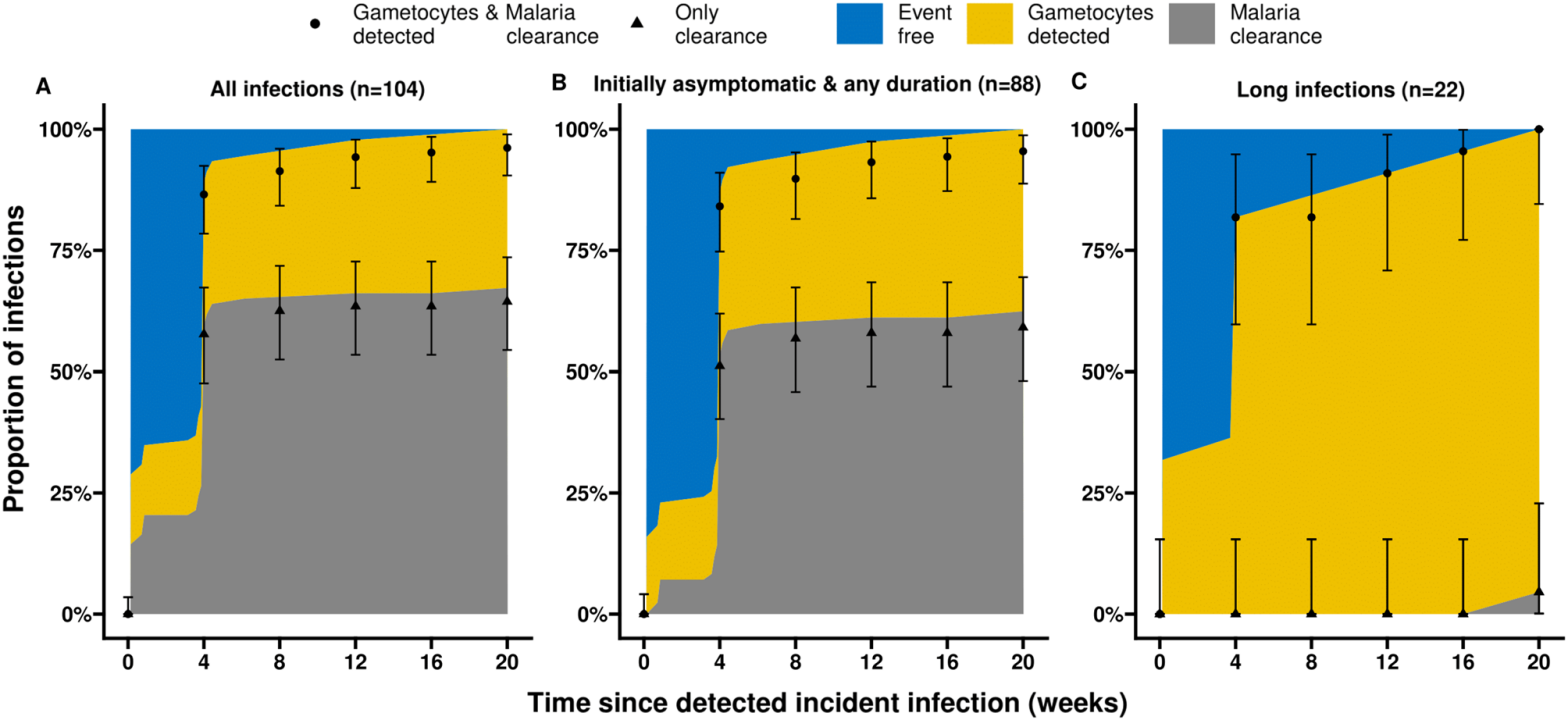
First detection of gametocytes and malaria infection clearance over time. These stacked plots show the Aalen–Johansen estimated cumulative proportion of infections that either carry detectable gametocytes (yellow) or resolve their malaria without detectable gametocytes over time -in weeks- (grey). Shown in blue is the proportion of infections that are event free over time, i.e. that have a detectable infection without detectable gametocytes. (**A**) Includes all 104 incident infections, symptomatic and asymptomatic. The percentage of infections having detectable gametocytes at the time of initial parasite detection (0 weeks) and by 4 weeks was 14% and 29%, respectively. (**B**) Includes 88 infections that were initially asymptomatic. The percentage of infections having detectable gametocytes between 0 and 4 weeks was 15.9% and 33.7%, respectively. (**C**) Includes 22 infections that were initially asymptomatic and had long duration of infection ($\ge$ 12 weeks). The percentage of long-duration infections that had detectable gametocytes at 0 weeks, between 0–4 weeks and 0–12 weeks was 31.8%, 81.8% and 90.9%, respectively. Triangles represent observed cumulative prevalences of malaria infection clearance over time; dots represent the summed cumulative prevalences of both the first detection of gametocytes and malaria clearance (to compare with the corresponding estimates in the stacked plot). 95% confidence intervals are presented in error bars. Right-censored infection times ((infections where gametocytes were not detected or malaria clearance was not observed) were not accounted for in the numerator of the observed prevalences, hence the underestimation in the cumulative incidence compared with the stack plot in (**A**) and (**B**). Long infections (**C**) did not have any right-censored observations.

Among all 104 incident infections, 14.4% (15/104) had gametocytes by qRT-PCR at the first moment their infection was detected by qPCR. Three asymptomatic infections had missing RNA samples at this time-point; gametocyte prevalence was 6. 3% (1/16) among infections that were initially symptomatic and 16.5% (14/85) among infections that were initially asymptomatic. Competing risks models were used to model time from first detection of an incident malaria infection by qPCR to the first detection of gametocytes by qRT-PCR (n = 33) while accounting for infection clearance without gametocyte detection (n = 67) and right-censoring (infections that neither carried detectable gametocytes nor cleared throughout follow-up, n = 4). Competing risk models (described in detail in the methodology section) model the time-to-event for a primary event (first detection of gametocytes) while accounting for a secondary event (parasite clearance) that prevents the primary event from being observed. If parasite clearance is not considered as a competing event then the cumulative incidence for detection of gametocytes is overestimated by the standard Kaplan–Meier method for time-to-event data. Additionally, we accounted for right-censoring. Right-censored observations are observations where partial data are observed in terms of the duration of infection. The data are incomplete because neither of the events are observed and we only know that the infection was event free (here: no detectable gametocytes were observed and infection clearance was not observed either) from the detection of incident infection until the last follow-up visit^34^*.* In this methodology right-censored observations contribute risk to both event types (first detection of gametocytes or infection clearance without detection of gametocytes); ignoring right-censoring (as in observed proportions) will result in underestimated cumulative incidences. We observed that the likelihood of having gametocytes detected prior to infection clearance was strongly dependent on the duration of infection. Among initially asymptomatic infections with an infection duration ≥ 12 weeks, 31.8% (7/22) had detectable gametocytes at the time of initial parasite detection, while 81.8% (18/22) and 90.9% (20/22) of infections had detectable gametocytes between 0–4 and 0–12 weeks after the infection was detected, respectively. When accounting for right-censoring in these long-duration infections, an estimated 83.3% had detectable gametocytes by 4 weeks after first detection of infection and 96% had detectable gametocytes by week 12 (Fig. 2). Although appearance of gametocytes was thus nearly universal among long-duration infections, these infections were a minority. When considering all initially asymptomatic infections regardless of their duration, only 36.3% (32/88) had detectable gametocytes prior to clearance; 4.5% (4/88) of infections were censored because they neither had detectable gametocytes nor resolved before study follow-up ended. Accounting for censoring, 29.5% of all infections and 33.7% of all initially asymptomatic infections had detectable gametocytes within the first month after the infection was detected. Thus, unless gametocytes were detected in the first 28 days, most infections (62.4%) did not have gametocytes at our level of detection prior to infection clearance (Supplemental Fig. S2). This estimate does not take into consideration that we sampled every 4 weeks and thus gametocytes that appeared and disappeared within this time-window may have been missed.

### Gametocyte density peaks within 4 weeks after infection detection

**Figure 3:**
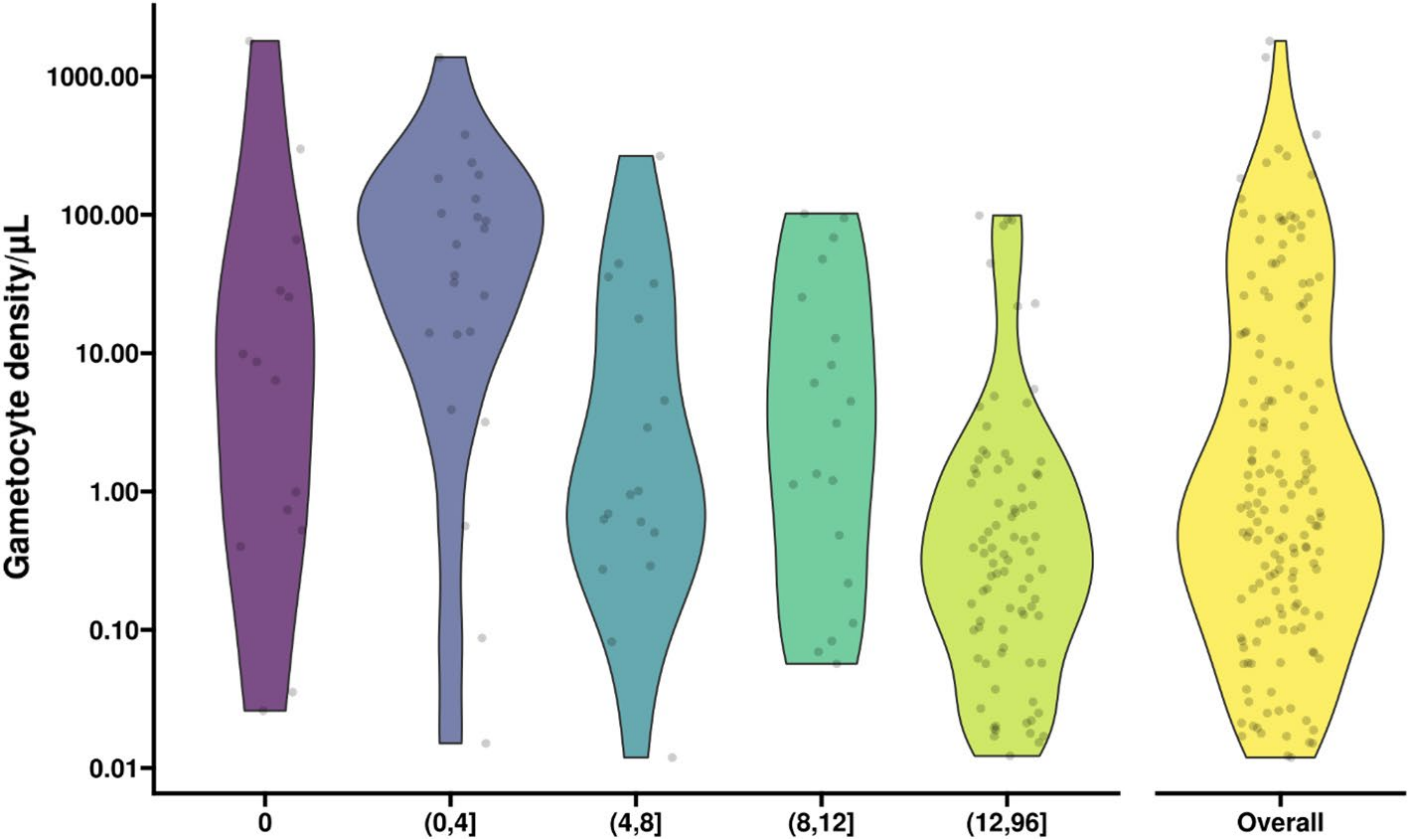
Gametocyte densities at different time intervals during infections. On the x-axis time in intervals is presented: T = 0 moment of infection detection (purple), 0–4 weeks (blue), 4–8 weeks (petrol), 8–12 weeks (green), 12–96 weeks (light green) post infection detection. On the y-axis are presented the observed gametocyte densities per µl. Overall gametocyte density (i.e. over the entire duration of follow-up) is presented in yellow. Gametocyte densities are presented for initially asymptomatic and gametocyte positive observations only (i.e. 32 incident infections with 155 gametocyte positive visits across them). Multiple observations from the same infection (multiple dots) may be included in the same violin plot if there were more than one gametocyte positive visit in the interval. Dots indicate raw data. The number of observations for each of the violin plots was: 14 (t = 0), 22 (t = 0–4), 17 (t = 4–8), 19 (t = 8–12), 83 (t = 12–96) at 155 (overall). Time points following parentheses are not included in the interval while those directly before square brackets are included. So the blue violin plot (0, 4] includes values from time 0 + 1 (not including 0) up to and including week 4.

Gametocyte densities may peak at different time-points following infection detection. The geometric mean of peak gametocyte densities was 14.33 gametocytes per µl (IQR: 2.56–97.68) among incident asymptomatic infections at first detection. When analyzing the dynamics of gametocyte densities over the course of infections, we observed that 28.1% (9/32) of peak gametocyte densities occurred at the initial visit, 50% (16/32) occurred between initial visit and week 4, and 21.9% (7/32) occurred at a timepoint beyond week 4. This temporal pattern of peak gametocyte densities in the first 4 weeks after infection detection, followed by declines in gametocyte densities, was also apparent when average gametocyte densities were plotted per time-interval following infection detection: at the initial visit, 14 initially asymptomatic parasite infections had a geometric mean of 4.61 gametocyte per µl (CI 95% 0.72, 29.51; IQR: 0.58–27.57); this rose to 23.81 (95 CI% 7.08, 80.12; IQR: 13.73–123.47; n = 22) gametocyte per µl for observations within the first 4 weeks after infection detection and subsequently declined to a density of 0.39 gametocyte per µl (95% CI 0.24, 0.63; IQR: 0.10–1.35; n = 83) across the 12–96 week period post infection detection (Fig. 3).

**Figure 4:**
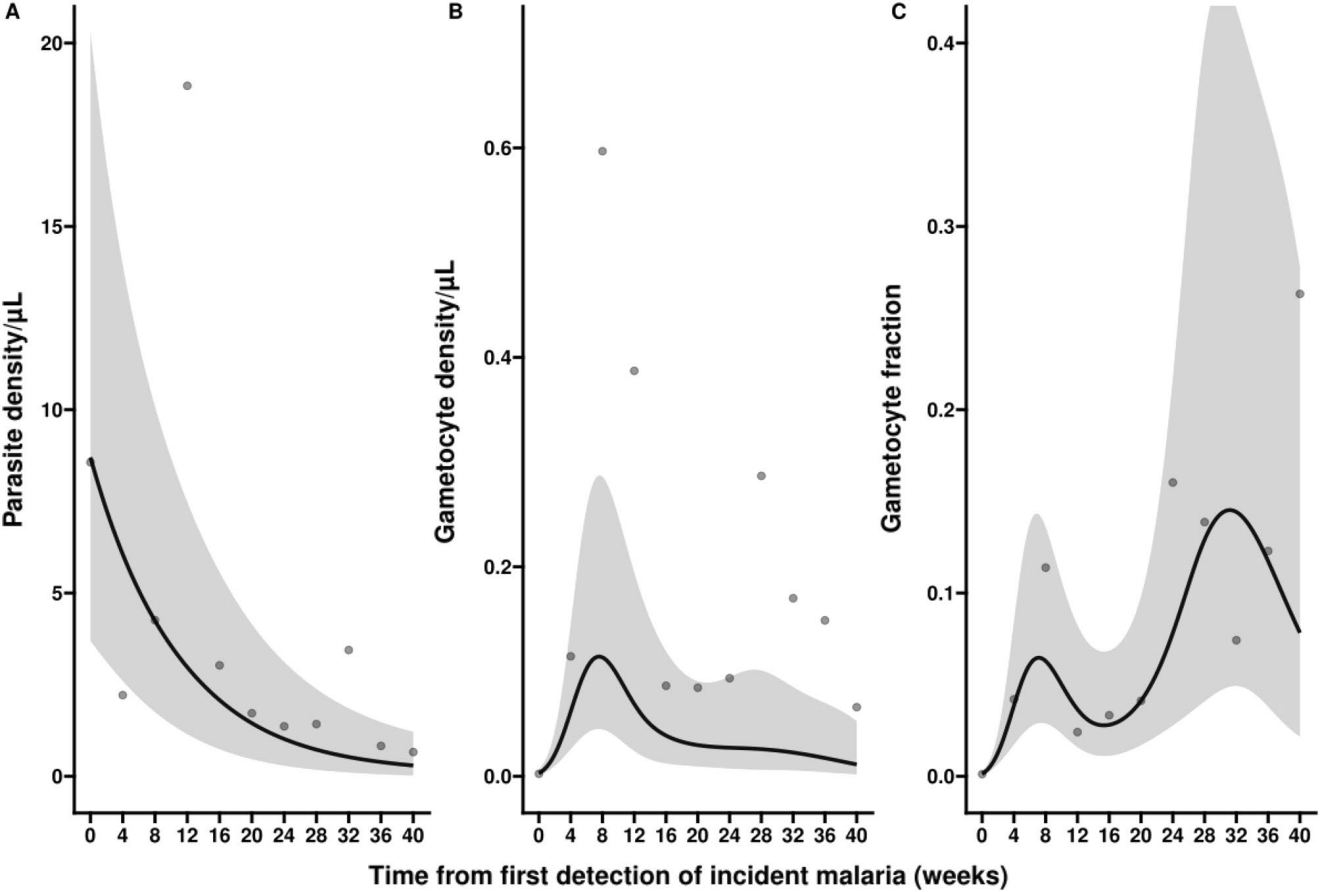
Parasite density, gametocyte density and gametocyte fraction over the course of incident infections. Three separate characteristics of infections are presented: parasite density (**A**), gametocyte density (**B**) and gametocyte fraction (**C**). Gametocyte fraction is defined as the proportion of parasites that are gametocytes, estimated as the proportion of the total parasite biomass (i.e. varATS parasite density by qPCR. Since this assay targets DNA and thus not discriminate between life-stages, this reflects the combined density of asexual parasites and gametocytes) that consists of gametocytes (estimated by gametocyte-specific qRT-PCR that targets Ccp4 and PfMGET mRNA). All estimates are presented over time since first detection of incident infection; all associations are best described by non-linear mixed-effects models, where a random intercept is used to account for correlation between measurements from the same infection. The dots represent the raw geometric mean parasite densities averaged after the previous timepoint and up to and including the timepoint where they are indicated (i.e. the dot at week 8 represents all values from week 4 + 1 day up to (and including) week 8). (**A**) The y-axis shows the estimated parasite density; on x-axis the time from first detection of infection presented in weeks. Parasite density is, on average, at highest levels at the detection of incident malaria and it rapidly declines until _~_ 6 weeks after detection, thereafter a slow and steady decline is observed. (**B**) Gametocyte densities are, on average, very low at first detection of incident malaria infections, but rapidly increase until 8 weeks later, thereafter a steady decrease is observed. (**C**) Over the course of an infection an increasing trend is seen for the ratio of gametocyte density over parasite density. An estimated decrease is seen at later time-points but this trend is highly uncertain, reflected in wide confidence intervals which also support an increase or a plateau.

Interestingly, the decline in asexual parasite densities did not mirror that of gametocyte densities. While asexual parasite densities declined immediately following the first detection of infection, followed by a more gradual decline in density after the first 4 weeks of infection (Fig. 4A), gametocyte densities peaked later (around 4–8 weeks after infection detection) and showed a gradual decline as infections persisted (Fig. 4B). As a consequence of these diverging kinetics, the proportion of parasites that were gametocytes increased over the course of infections (Fig. 4C).

### Gametocyte appearance is dependent on parasite density, multiplicity of infection and sickle cell trait

**Figure 5:**
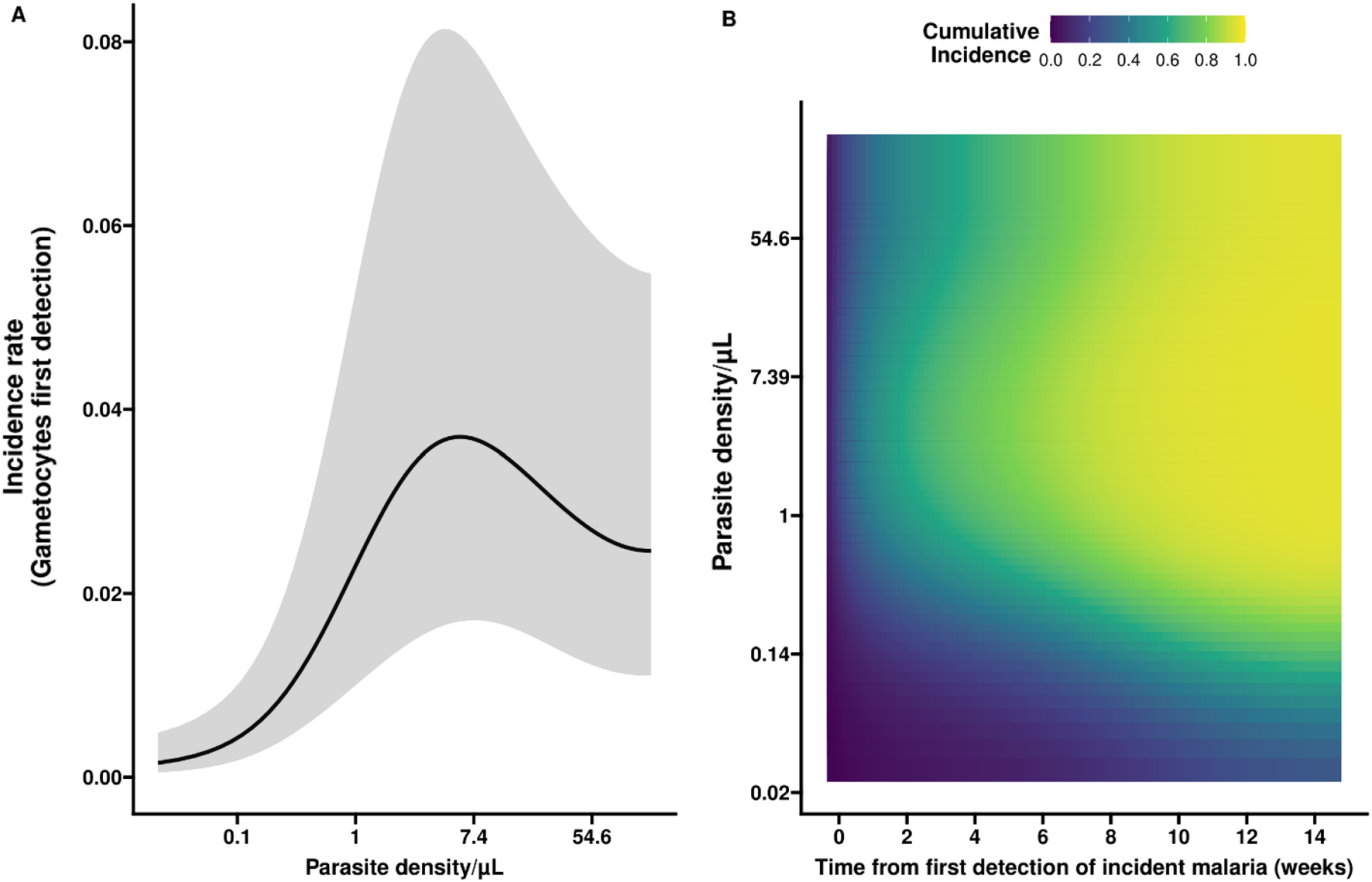
First detection of gametocytes in relation to parasite density and infection duration. Panel (**A**) shows the non-linear association between parasite density/µl (natural log (ln) transformed axis) and the incidence of detectable gametocytes (per person-day), adjusted for duration of infection. Higher parasite densities are associated with higher gametocyte incidence rates up to parasite densities of ~ 5 parasites/µL. While the association is seemingly negative after this point, the wide confidence intervals (grey shaded areas) indicate high levels of uncertainty. Panel (**B**) presents the association between parasite density and the duration of incident infection with the cumulative incidence of gametocyte positivity. In (**B**), on the y axis the parasite density is expressed per µl on a natural log (ln) transformed axis, while the x axis describes the time in weeks since the first detection of infection. Low cumulative incidence is shown in blue, whilst cumulative incidences closer to 100% are shown in yellow. Expected cumulative incidences for gametocyte positivity are presented over the full duration of an infection if parasite densities would be maintained at the level given on the y-axis; the likelihood of having detectable gametocytes increases with increasing parasite density and longer time since infection.

Detection of gametocytes during follow-up was positively associated with parasite density (Fig. 5A). In our cohort, this association was apparent up to a parasite density of ~ 5 parasites/µL while observations from higher parasite densities were sparser and we could not reliably determine whether this positive association continued. An apparent plateau in the association between parasite density and the incidence of detectable gametocytes may be explained by symptomatic infections that are associated with high parasite densities but low gametocyte incidence due to interruption of the infection by antimalarial treatment. Figure 5B illustrates the association between the first detection of gametocytes (cumulative incidence of gametocyte positivity) and parasite density. For this, it is assumed that parasite densities are maintained at constant density over the course of the infection. If an infection maintains parasite densities above a certain threshold, e.g. at 1 parasites/µL, over time, we expect nearly all incident infections to have had detectable gametocytes by 8–10 weeks after detection. The pattern demonstrates that if lower parasite densities are maintained over the course of infection, as was often the case in our cohort, the likelihood of detecting gametocytes over the course of this infection was similarly reduced.

**Figure 6:**
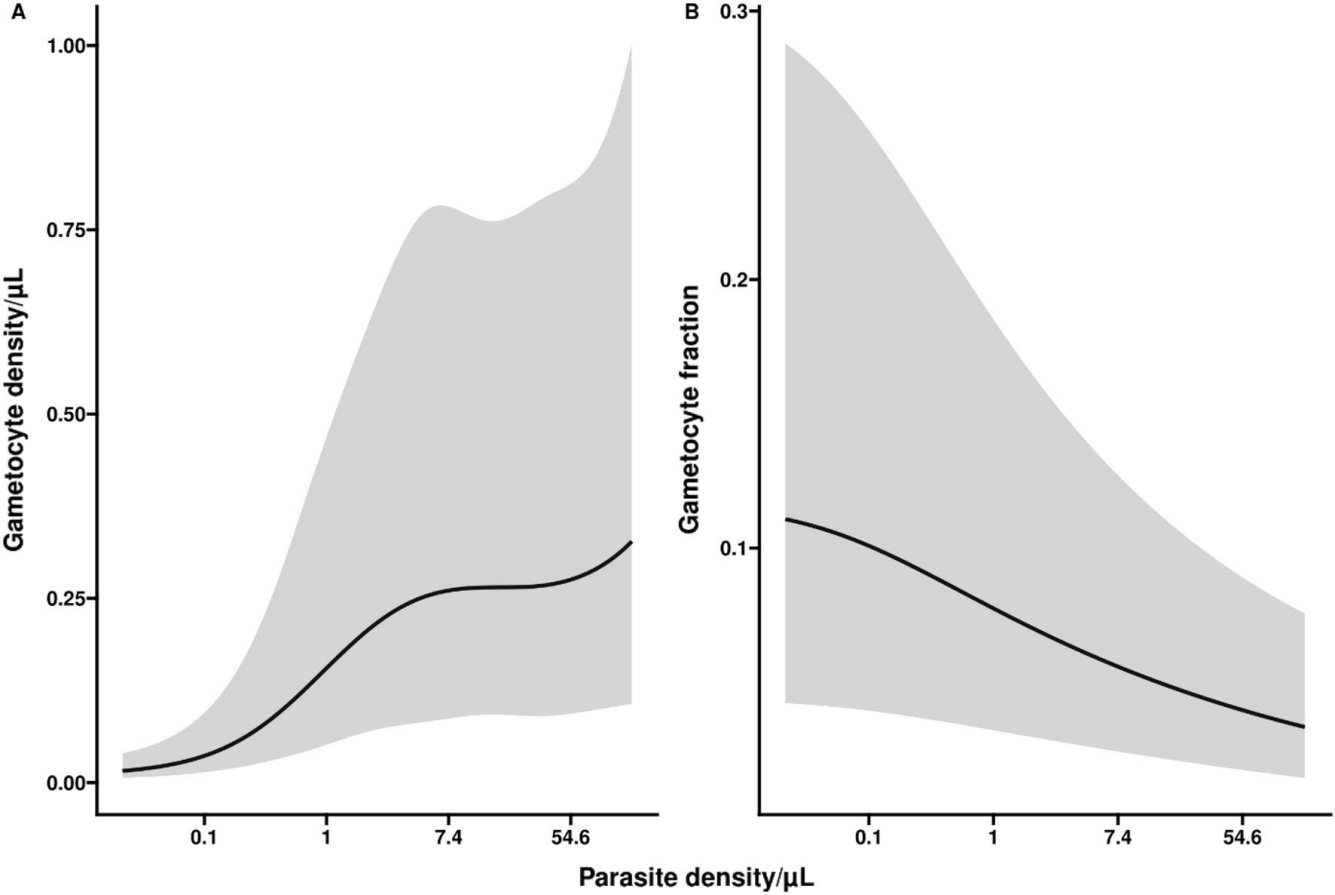
Gametocyte density and gametocyte fraction in relation to parasite density. Parasite density/µl is plotted in natural log (ln) scale within the range of the data. In panel (**A**), the association of parasite density with gametocyte density is presented, adjusted for the duration of infection. We observe a positive association between total parasite density and gametocyte density. In panel (**B**), the association of parasite density with gametocyte fraction is presented, adjusted for the duration of infection. Higher parasite densities are associated with lower gametocyte fraction at any given moment in time. Visits when the infection was detected without detectable gametocytes were included in these analyses.

Parasite density was positively associated with gametocyte density but negatively associated with the proportion of parasites that was gametocyte (Fig. 6). This pattern remained apparent when excluding symptomatic infections (Supplemental Table S1). Adjusted for parasite density, gametocyte appearance and gametocyte densities over time were associated with several host characteristics. Although not statistically significant, gametocytes were 1.93 times (95% CI 0.71, 5.23; p < 0.1853) more likely to be detected during infections in children aged 5–15 years compared to children < 5 years of age (Table 2)*.*

**Table 2:** Associations of parasite metrics with human host and infection characteristics.

	Parasite density		Incidence of detectable gametocytes		Gametocyte density		Gametocyte fraction				Malaria resolution			
	DR (95% CI)	p-value	HR (95% CI)	p-value	DR (95% CI)	p-value	DR (95% CI)		p-value		HR (95% CI)			p-value
Age (< 5 years = reference group)														
5–15 years	0.72 (0.09, 5.87)	0.7505	1.93 (0.71, 5.23)	0.1853	1.46(0.38, 5.68)	0.5777	1.09 (0.35, 3.38)		0.8727		0.82 (0.43, 1.58)			0.5471
16 + years	0.12 (0.01, 1.13)	0.0589	1.16 (0.38, 3.54)	0.7835	0.79 (0.19, 3.33)	0.7447	1.12 (0.34, 3.73)		0.8462		0.24 (0.12, 0.49)			0.0001
Gender (female = reference group)														
Male	0.5 (0.09, 2.71)	0.4133	0.97 (0.45, 2.09)	0.9402	1.11 (0.39, 3.17)	0.845	1.24 (0.53, 2.89)		0.6165		0.75 (0.43, 1.31)			0.3055
HB genotype (HbAA = reference group)														
HbSS	8.33 (0.45, 154.16)	0.1464	1.04 (0.21, 5.05)	0.9637	1.53 (0.23, 10.11)	0.6512	1.55 (0.31, 7.88)		0.5869		1.44 (0.61, 3.39)			0.3952
HbAS	1.69 (0.22, 13.11)	0.6083	2.68 (1.12, 6.38)	0.0231	9.19 (2.79, 30.23)	0.0002	3.13 (1.14, 8.59)		0.0241		0.43 (0.19, 0.96)			0.036
Clonality (monoclonal infection = reference group)														
Polyclonal	15.43 (2.62, 90.98)	0.002	2.93 (1.16, 7.44)	0.0208	5.96 (1.4, 25.32)	0.0135	2.45 (0.76, 7.87)		0.1255		0.40 (0.17, 0.91)			0.0265

This table describes the effect of host (age, gender, Hb polymorphism) and parasite characteristics (clonality) on the main outcome measures parasite density, incidence of detectable gametocytes, gametocyte density, gametocyte fraction and the incidence of infection clearance without gametocyte detection (malaria resolution) with respect to their temporal patterns since infection detection. The hazard ratios (HRs) are used to summarize the competing risks modelling of time to two possible events: first detection of gametocytes or time to parasite clearance without gametocyte detection. For each event and across time, the hazard rate is defined as the rate at which an infection experiences the event at some time conditional on the infection being event-free before that time. Incidence of detectable gametocytes is thus the rate at which gametocytes are first detected in infections and malaria resolution is the rate at which infections clear without gametocyte detection. The HR for a covariate summarizes how much higher/lower the hazard rate over time is for a covariate value relative to a baseline value for that covariate. The density ratios (DRs) are used to summarize the non-linear mixed-effects modelling of the longitudinal trajectories of parasite density, gametocyte density and gametocyte fraction over time. The DR for a covariate, similar to HR, summarizes how much higher/lower the densities over time is for a covariate value relative to a baseline value for that covariate.

Moreover, participants 16 years and older were less likely (HR: 0.24, 95% CI 0.12, 0.49; p = 0.0001) to resolve their infection before gametocytes were detected, when compared with children under-five. Notably, gametocyte appearance was over two times more likely (Hazard Rate (HR) = 2.68, 95% CI: 1.12, 6.38; p = 0.0231) among infections in individuals with the HbAS compared to the HbAA genotype. In contrast, no association was observed for the limited number of infections with HbSS in our cohort (n = 10; HR = 1.04, 95% CI 0.21, 5.05; p = 0.9637). These findings persisted amongst infections that were initially asymptomatic (n = 88) and amongst the 22 long infections (Supplementary Fig. S3). Infections among individuals with the HbAS genotype also had higher gametocyte densities over the duration of infection compared to those with HbAA (Density Ratio (DR) = 9.19, 95% CI 2.79, 30.23; p = 0.0002). This elevated gametocyte density for HbAS compared to HbAA greatly exceeded differences between these populations in total parasite density (Table 2) and resulted in a threefold increase in gametocyte fraction for infections in HbAS, compared to HbAA individuals (DR = 3.13, 95% CI 1.14, 8.59; p = 0.0241). The same trend was seen when comparing peak gametocyte densities that were higher in infections in individuals with the HbAS genotype (73.59 gametocytes/µL, IQR: 31.32–189.12) compared to those with HbAA (4.97 gametocytes/µL, IQR: 0.51–29.29; p = 0.0060) and when excluding symptomatic infections at the first moment of parasite detection (Supplemental Table S1). Among infectious individuals, 23.8% (5/21) had the HbAS genotype and 9.5% (2/21) had HbSS genotype. There was no obvious difference in the association between gametocyte density and mosquito infection rate between HbAA and HbAS/HbSS individuals (Supplemental Fig. S4).

Regarding infection characteristics, polyclonal infections had higher asexual parasite densities (DR = 15.43, 95% CI 2.62, 90.98; p = 0.002)) and gametocyte densities (DR = 5.96, 95% CI 1.4, 25.32; p = 0.0135) (Table 2). As a result, there was no statistically significant increase in gametocyte fraction in polyclonal infections (DR = 2.45, 95% CI 0.76, 7.87; p = 0.1255). Further supporting the role of HbAS and polyclonal infections in gametocyte appearance, we observed that infections among individuals with the HbAS genotype or polyclonal infections were less likely to resolve their infection prior to first detection of gametocytes (HR = 0.43, 95% CI 0.19, 0.96; p = 0.0360 and HR = 0.40, 95% CI 0.17, 0.91; p = 0.0265, respectively); these associations remained significant after adjusting for parasite density at first detection of infection (Supplemental Table S2). The limited number of incident infections with information on the complexity of infection, made it impossible to explore the effects of sickle cell status and polyclonal infections concurrently in a multivariate model.

### Sensitivity analysis: the associations of gametocyte appearance with HbAS and polyclonality were not driven by incident infections with very low parasite densities

Some of the associations presented above may be influenced by parasite density that is associated with both infection duration and the detectability of gametocytes and parasite clones. To explore the impact of this factor, we performed a sensitivity analysis by removing all 29.8% (31/104) of infections with an initial parasite density below 0.1 parasites per µl and re-running the models (Supplemental Table S2). In this selection, there were 17/73 HbAS individuals (23.3%, compared to 21.8% in the complete dataset), 6/73 HbSS individuals (8.2%, compared to 8.9% in the complete dataset and 25/66 polyclonal infections (37.9%, compared to 37.3% in the complete dataset) (Supplemental Table S3). Using this selection for the sensitivity analysis, the temporal patterns of parasite density, gametocyte density and gametocyte fraction remained the same but, as a logical consequence of this selection of higher parasite densities, there was a shift in the positive direction on the y-axis for parasite density and gametocyte density (not for gametocyte fraction) (Supplemental Fig. S5). The association between parasite density and incidence of detectable gametocytes showed a similar trend to Fig. 5, with a more apparent plateau (Supplemental Fig. S6). Similarly, the positive association between parasite density and gametocyte density and the negative association between parasite density and gametocyte fraction remained apparent after excluding infections with low initial parasite densities (Supplemental Fig. S7). The effects of HbAS and multiplicity of infection were similarly consistent (Supplemental Tables S2, S3).

### Sensitivity analysis: the association of multiplicity of infection (MOI) with gametocyte density may be driven by symptomatic infections

A further sensitivity analysis was performed. Here we removed all 27% (28/104) of infections that were symptomatic at any time. In this selection, there were 18/76 HbAS individuals (24%, compared to 21.8% in the complete dataset), 4/76 HbSS individuals (5.3%, compared to 8.9% in the complete dataset) and 12/39 polyclonal infections (30.8%, compared to 37.3% in the complete dataset) (Supplemental Table S4). Using this selection for the sensitivity analysis, the temporal patterns of parasite density, gametocyte density and gametocyte fraction continued to be the same but, as expected, parasite and gametocyte densities were much lower (Supplemental Fig. S8). The association between parasite density and incidence of detectable gametocytes showed a similar trend to Fig. 5 (Supplemental Fig. S9), albeit with much lower cumulative incidences (driven by the lower parasite and gametocyte densities). The positive association between parasite density and gametocyte density and the negative association between parasite density and gametocyte fraction persisted (Supplemental Fig. S10). HbAS was still statistically significantly associated with detection of gametocytes and gametocyte density, while the effect of having a polyclonal infection on gametocyte density no longer achieved statistical significance (Supplemental Table S2).

### Sensitivity analysis: accounting for interval-censoring increases uncertainty in the association of multiplicity of infection (MOI) with gametocyte appearance while the effect of HbAS persists

In addition to the findings of both the above sensitivity analyses, we found through a further sensitivity analysis that included all 104 infections that, when accounting for interval-censoring, the effect of polyclonality on time to first detection of gametocytes may no longer be statistically significant. This interval-censoring was considered important since the exact time at which the incident malaria infection, first detection of gametocytes, or infection clearance occurred was not known due to the nature of routine sampling every 4 weeks. Ignoring interval-censoring may lead to underestimated standard errors and thus inflated type I error rates, i.e. false positive findings. We investigated how sensitive the results of our modelling were to the interval-censored nature of the event times, by imputing exact times using a uniform distribution from the intervals in which they were observed. We imputed one hundred datasets and re-ran the models for all outcomes. For the time to first gametocyte detection, HbAS showed statistical significance (p < 0.05) in 81% of the imputed data, and the remaining 19% were significant at a 10% threshold for significance (p < 0.10) (Supplementary Fig. S11B plot). The distribution of the effects (HRs) for HbAS compared with HbAA from the imputed data (Supplementary Fig. S12B plot) appeared to correspond well with the effect of HbAS from the main findings (i.e. without considering interval censoring). Conversely, polyclonality was only statistically significant (p < 0.05) in 10% of the imputed data, with a wider range of p-values (Supplementary Fig. S11B plot). Further, the distribution of the effects for polyclonality from the imputed data appeared noticeably lower but these hazard ratios were closer to 2 than to 1 (where 1 indicates no effect) (Supplementary Fig. S12B plot). With a large proportion of missingness for multiplicity of infection, and increased variability when considering interval censoring, it is difficult to draw any meaningful conclusion on the magnitude or statistical significance of the observed effects; we only conclude that there are indications for an association of polyclonality of infection with faster gametocyte appearance and higher gametocyte density but not with parasite density or gametocyte fraction. For the longitudinal modelling of parasite density, gametocyte density and gametocyte fraction, we see a nearly unanimous agreement across imputations indicating a statistically significant effect of HbAS on gametocyte density and gametocyte fraction (Supplementary Fig. S11C,D) and a significant effect of polyclonality on parasite density and gametocyte density (Supplementary Fig. S11A,C). The distribution of the effects (DRs) from the imputed data (Supplementary Fig. S12) for all outcomes (A–D plots) closely align with results from the main study findings (Table 2). None of the imputations showed a significant effect of HbAS on parasite density (Supplementary Fig. S11A) or of multiplicity of infection on gametocyte fraction (Supplementary Fig. S11C,D), as we had expected from our main findings.

## Discussion

Here, we longitudinally monitored 104 infections acquired in an area with a low force of infection but historically exposed to intense malaria transmission. We observed that incident infections, that occurred in all age groups, were typically of very short duration and commonly did not have detectable gametocytes over the course of infection. In the minority of asymptomatic infections that persisted for 3 months or longer, gametocyte appearance was near universal with earlier gametocyte detection and higher densities in infections with higher total parasite densities and among individuals with the HbAS genotype.

Infections with *P. falciparum* can be of very long duration. In our cohort, some individuals carried infections for 2 years or longer^33,35^ and infections of up to 14 years have been documented elsewhere^15^. Because chronic infections are considered important sources of transmission^9^ and plausible hurdles for malaria elimination initiatives^15^, they understandably received considerable attention. Our detailed parasitological monitoring with sensitive molecular diagnostics, uncovered that only a minority of incident bloodstream infections result in chronic parasite carriage. These long infections are important in driving transmission whilst, our findings demonstrate, many other infections may be relatively unimportant. There are several previous reports on the occurrence of brief episodes of parasitemia; these are typically based on microscopy and leave uncertainties about possible persistence of parasitemia at submicroscopic densities (reviewed in^14^). Using a highly sensitive qPCR^36^ in cohort participants who were initially free of infection, we identified 104 incident infections, 88 of which were initially asymptomatic and observed that 56% (49/88) of these incident initially asymptomatic infections were observed only once. The exact duration of these infections was impossible to estimate with precision, given the 4-week interval between scheduled visits, but it is evident that the majority of infections were brief and spontaneously cleared. While naturally acquired immunity has been suggested as determinant of infection clearance^37^, recent analyses suggest that infection duration may be shortest in young children^35^ and acquired immunity may actually be a prerequisite to sustain chronic infections^38^, rendering a role of immunity-dependent infection clearance less obvious. Importantly, we provide the first evidence that these short-duration infections do not result in measurable gametocyte densities. It is possible that very brief episodes of gametocytemia were missed and this could have increased the relative importance of short-duration infections for transmission. However, we consider it unlikely that this was common, given that gametocytes typically persist for approximately one month after infections are cleared with gametocyte-permissive drugs in controlled human infections^39^. It is, however, conceivable that very low gametocyte densities have been missed. The majority of infections that were of short duration were of very low density and since gametocyte densities comprise only a small proportion of the total parasite biomass, very low gametocyte densities are plausible in these infections and may have remained undetected. The strong association we report between asexual parasite density and the appearance of detectable gametocytes further demonstrates that the detectability of gametocytes is closely linked to that of their asexual progenitors. Our findings are therefore not contradicting the notion of stochastic gametocyte commitment in a minority of parasites^40^ but rather demonstrate that gametocyte densities that are most likely to result in onward transmission to mosquitoes, above ~ 5 gametocytes/µL^33^, are not achieved by the majority of incident infections. Our findings further demonstrate that stochastic commitment to gametocyte production may only be one of the determinants gametocyte carriage and also human and infection characteristics may play a role^18,21^. We observed strong indications for increased gametocyte production in individuals with sickle cell trait (HbAS). HbAS has previously been associated with lower parasite densities and a reduced chance of clinical disease upon infection^27,41^. Moreover, also an increased transmission potential of HbAS parasite carriers has been suggested before^24,31^. Our study is the first to relate the appearance of gametocytes to HbAS in longitudinally monitored infections. We observed that the first appearance of detectable gametocytes was earlier and gametocyte densities over the course of infections were higher for infections in individuals with the HbAS genotype as previously observed in individuals with the HbAC genotype^23^. Moreover, the proportion of parasites that were gametocytes was higher, suggestive of higher per-parasite gametocyte production. More frequent sampling is required to formally demonstrate differences in gametocyte production but it is conceivable that gametocyte formation is triggered in HbAS blood cells where parasite growth is reduced^42^ and erythrocyte sickling upon parasitization is increased^43^. Our previously reported assessment of gametocyte infectivity^33^ allowed us to explore the viability of gametocytes in infections with HbAA and HbAS. Although data were too sparse for detailed analysis, the representation of different HbS genotypes among infectious individuals (n = 21) was similar to that of the entire population (Table 1). Whilst we found no evidence to support previous claims of higher gametocyte infectivity in HbAS^31^, our mosquito feeding assays give us confidence that our observation of higher gametocyte production translates in an increased transmission potential for infections in individuals with the HbAS genotype. This increased transmission potential will be enhanced by the fact that infections also last longer in HbAS and delayed time to clinical symptoms might further favor gametocyte development^27,44^. Controlled human malaria infection (CHMI) studies have confirmed prolonged time to infection detection in HbAS individuals^41^. With the development of CHMI studies that permit gametocyte development^45^, it is conceivable that our indications for enhanced gametocyte formation and previous reports on increased infectivity in HbAS individuals can be further quantified in a controlled setting. Given the high HbS allele frequencies in sub-Saharan African countries and their potential for carrying infective gametocytes, more attention should be given to their contribution to the infectious reservoir. Asymptomatic individuals with sickle cell trait might represent a source of transmission that is difficult to identify in population surveys.

The presence of competing parasite clones is also considered as trigger for increased gametocyte formation^46,47^. Our findings of higher gametocyte densities in polyclonal infections appear supportive of these associations but nevertheless have to be interpreted with caution. Although patterns persisted in a sensitivity analysis where the lowest-density infections were excluded, it remains possible that the association is (partly) driven by a higher detectability of parasite clones in higher density infections that are also associated with higher gametocyte production.

Our study had several limitations. The exact time of incident infection, and gametocyte initiation was not known. The generalized additive mixed modelling (GAMM) framework^48,49^ that we used to model the non-linear trajectories of parasite density, gametocyte density, incidence of detectable gametocytes and gametocyte fraction over time, does not account for the uncertainty surrounding observation of the exact times. Here, duration of infection was defined as the time from first detected incident infection and for the time-to-event models, the event time was defined as the time from the first detected incident infection to first detected gametocytes. To investigate how sensitive our model was to the interval censored nature of the incident infection, and also for the start of gametocyte appearance and infection resolution (for the time-to-event model), we imputed 100 datasets with times uniformly generated from within their intervals and observed consistency in our reported associations between gametocyte metrics and HbAS and polyclonality of infections. As a result of the limited number of infections and failure to determine the complexity of infection in a substantial number of infections, especially in low-density infections^54^, we were unable to assess the impact of covariates on gametocyte production in a multivariable model.

Additionally, we used a single genetic marker (AMA-1) coupled with amplicon deep sequencing to detect different haplotypes. Although it is possible that our estimated multiplicity of infection would have been higher if we had used multiple targets^55^, the diversity of AMA1 was quite high (He = 0.949). This implies a low chance (~ 5%) of infection with the same clone^35^ and failure to detect all (incident) clones is thus unlikely to have affected our main associations.

In conclusion, this study provides evidence that a large proportion of asymptomatic infections in all age groups are of short duration and low parasite density, lasting less than a month, and not associated with gametocytemia. Although in minority, long lasting chronic asymptomatic infections are characterized by high gametocyte densities and transmission potential and infection duration and gametocyte production were influenced by host genetic status. These findings, from an area where intensive mosquito control which was exceptionally effective in driving down transmission, are of relevance for malaria elimination initiatives. Such initiatives have to consider that whilst chronic asymptomatic infections form a major reservoir for infectious gametocytes, this status is only achieved by a minority of incident infections.

## Material and methods

### Study site

Data were obtained from a prospective cohort study conducted in Nagongera subcounty, Tororo district near the Kenyan border between October 2017 and October 2019^56^. Historically, this was an intense transmission setting where malaria transmission was highly reduced following repeated long-lasting insecticidal nets (LLIN) distributions and sustained and effective and indoor residual spraying of insecticides (IRS)^56^. 531 participants were recruited from 80 randomly selected households as described previously^33^. Ethical approval for the study was obtained in Uganda, Uganda National Council for Science and Technology (UNCST: HS119ES), School of Medicine Research and Ethics Committee (SOMREC: 2017-099), United Kingdom, London School of Hygiene and Tropical Medicine (LSHTM: 14266), and United States, University of California San Francisco (UCSF IRB: 17-22544), Stanford (UCSF IRB reliance).

The study was performed in accordance with the Declaration of Helsinki as well as the “International Council for Harmonization of Technical Requirements for Pharmaceutical for human Use-Good Clinical Practice (ICH-GCP)”guidelines. Informed consent was collected from all cohort participants prior to enrollment in the study and/or their legal guardian(s) per IRB guidelines. At enrollment, blood samples were screened for sickle cell trait HbAS. Cohort study participants were encouraged to come to a dedicated study clinic open 7 days per week for all their medical care. Routine visits were conducted every 28 days and included a standardized clinical evaluation and collection of blood for thick blood smear, hemoglobin measurement (every 12 weeks), and storage for future molecular studies including ultrasensitive varATS qPCR and gametocyte quantification by reverse transcriptase qPCR targeting female (Ccp4) and male (PfMGET) gametocyte transcripts^57^. Study participants with fever (tympanic temperature > 38.0 °C) or history of fever in the previous 24 h had a thick blood smear read immediately. If the thick blood smear was positive by light microscopy, the patient was diagnosed with malaria and treated with 3-day course of artemether-lumefantrine (Coartem; Novartis, Basel, Switzerland). Participants with asymptomatic parasitemia were not given antimalarial treatment in accordance with national guidelines.

### Laboratory methods

200 µL whole blood samples were collected at enrollment, at routine each visit and at each clinical malaria episode^56^. DNA extraction was performed by Qiagen spin columns (Qiagen QIAamp DNA Blood Mini Kit) and 5 µl of extraction products was assessed for presence and quantification of *P.falciparum* parasites by varATS qPCR which has a limit of detection of 0.05 parasites/µL^58^. Samples with > 0.1 parasite/µl were genotyped by apical membrane antigen-1 (AMA-1) amplicon deep sequencing^35^.

For all qPCR positive samples, 100 µL of blood in RNA protect (RNA protect Qiagen), prior to DNA extraction, was used to quantify female (CCp4 mRNA) and male (PfMGET mRNA) gametocyte transcripts by quantitative reverse transcriptase PCR (qRT-PCR) with a lower limit of detection of 0.1 gametocytes/µL^59^.

### HBS genotyping

Genotyping of HbS mutation (dbSNP rs334) used molecular inversion probe (MIP) capture and deep sequencing on Illumina NextSeq 550^60^. MIP capture incorporated unique molecular identifiers (UMIs) allowing subsequent collapse of reads representing PCR duplicates of the same captured molecule. Individuals SNP genotype calls required ≥ 10 UMI depth and ≥ Q20 genotype quality to ensure capturing both alleles (99.8%) in a heterozygous individual with trait.

### Gametocyte outcome measures

We analyzed main outcome measures such as the incidence of detectable gametocytes and the incidence of infection clearance without gametocyte detection, parasite density, gametocyte density and gametocyte fraction in relation to time since infection and in relation to host and parasite characteristics. The incidence of detectable gametocytes describes the measure of the frequency of first detection of gametocytes over time, whilst the incidence of the infection clearance prior to gametocyte detection describes the frequency of infections that resolve without gametocytes ever being detected by molecular methods. The incidences of detectable gametocytes or infection clearance without gametocytes were measured through the hazard rate, defined as the likelihood of an event occurring over time conditional that it did not occur before. We also estimated the cumulative incidences, i.e. the proportion of infections that had detectable gametocytes before a certain time and the proportion of infections that cleared without gametocytes being detected by a certain time. This is different from the observed proportions, in that the observed proportions exclude right-censored observations entirely after the time in which they are right-censored. These right-censored observations were incident infections in which gametocytes were never detected and did not clear by the end of their follow-up. The gametocyte fraction was defined as the proportion of the total parasite biomass of parasites that was gametocyte.

### Statistical models

In this study, we modelled the non-linear trajectories of parasite density, gametocyte density, incidence of detectable gametocytes and gametocyte fraction over time using the generalized additive mixed modelling (GAMM) framework^48,49^. To model the effects of covariates, we were able to adjust for this non-linear effect of parasite density over time. GAMMs allows for the modelling of complex non-linear relationships using smoothing splines^50^ without the need for parametric assumptions of the shapes of the trajectories. GAMMs are extensively used in areas such as ecology^51^, and have been used to model other infectious disease processes with complex non-linear effects and interactions, such as seasonality^52^. While under-utilized in malaria epidemiology, Rodriguez-Barraquer^53^ used GAMMs to model the malaria incidence, parasite prevalence and density over age and exposure. They showed how the non-linear trajectories of malaria incidence and parasite prevalence over age varied across sites and they showed the complex bivariate effect of age and exposure on parasite density and the ‘fever threshold’.

### Incidence model: detection of gametocyte initiation over time since first detection of infection

The time from first detection of an incident infection with malaria parasites to the detection of gametocytes was studied applying theory from survival analysis. In survival analysis, this time-span is often modeled via the hazard function which is a function of time as well as covariates of interest (e.g. parasite characteristics, participant demographic or genetic characteristics). Here, we chose to model this hazard function as a piece-wise exponential additive model (PAM), a model within the GAMM framework, to have sufficient flexibility^61,62^. In the analysis, a competing risk needed to be accounted for since some incident infections may resolve before gametocytes are detected and these incident infections no longer contribute risk for detecting gametocytes. Therefore, we considered the *cause-specific* baseline hazards functions for time to first detection of gametocytes and for time to clearance of infection prior to gametocyte first detection. The parameters in the model were estimated by a penalized maximum likelihood method within a competing risks framework. More specifically, and also similarly described elsewhere^52,62–64^ the hazard function at time $t$ is defined as

1 $$\lambda \left(t|{x}_{i,q}\left(t\right),{x}_{i,p},k\right)=\mathrm{exp}\left({\beta }_{0,k}+{f}_{0,k}\left({t}_{j}\right)+{g}_{k}\left({x}_{i,q}\left({t}_{j}\right)\right)+{\beta }_{p,k}{x}_{i,p}\right) \forall t\in \left({\tau }_{j-1},{\tau }_{j}\right],$$

where events $k=1, 2$ indicate gametocyte initiation and clearance of infection prior to gametocyte initiation respectively, $\mathrm{exp}\left({\beta }_{0,k}+{f}_{0,k}\left({t}_{j}\right)\right)$ is the baseline hazard function at time ${t}_{j}$ for each of the events respectively, and ${t}_{j}$ is a chosen value of time within the interval (in our case ${t}_{j}={\tau }_{j}$, the right boundary) that was used to make the hazard depend on time while being constant over time within the interval. Note that other choices of ${t}_{j}$ within the interval $\left({\tau }_{j-1},{\tau }_{j}\right]$ can be made. Furthermore, ${g}_{k}(.)$ is the non-linear function for the effect of the time-varying covariate log parasite density $\left({x}_{i,q}(t)\right)$ for each of the $k$ events. Regression coefficient ${\beta }_{p,k}$ is the unknown coefficient for the effect of the ${p}^{th}$ covariate ${x}_{p}$ for each of the $P$ covariates. Non-linear functions are represented through basis functions and coefficients, e.g. ${f}_{0,k}\left(s\right)=\sum_{l=1}^{L}{\gamma }_{l,k}{b}_{l,k}(s)$ where ${\gamma }_{l,k}$ are unknown regression parameters to be estimated and ${b}_{l,k}\left(s\right)$ are chosen basis functions. We used B-spline basis functions^65^. A succinct introduction to splines, including B-splines, is provided by^50^. The PAM applied to the time intervals is equivalent to the Poisson GAMM where the effect of time is modelled with penalized splines stratified by event type, but without the random-intercepts component. This makes it possible to estimate the parameters in the model by their maximum likelihood estimates. To overcome overfitting a penalty was added to the log likelihood function.

We considered several models. We first fit a model with only a spline for time to visualize the baseline hazards for both events, gametocyte first detection and infection clearance prior to gametocyte detection, i.e. $\lambda \left(t|k\right)=\mathrm{exp}\left({\beta }_{0,k}+{f}_{0,k}\left({t}_{j}\right)\right)$. To visualize the (potentially non-linear) effect of parasite density as a time-varying covariate on first gametocyte detection, we fit a model with a penalized spline for the non-linear effect of log parasite density, i.e. $\lambda \left(t|{x}_{i,q}\left(t\right),k\right)=\mathrm{exp}\left({\beta }_{0,k}+{f}_{0,k}\left({t}_{j}\right)+{g}_{k}\left({x}_{i,q}\left({t}_{j}\right)\right)\right)$. To assess the effects of the remaining time independent categorical covariates, we fit independent models for each covariate as specified in Eq. (1).

### Non-linear models: parasite density, gametocyte density and gametocyte fraction over time since first detection of infections

To study the non-linear trajectories of the repeated measurements of parasite density, gametocyte density and gametocyte proportion (proportion of the total parasite biomass that is gametocyte) over the duration of infection we fit GAMMs for each of these log transformed outcomes respectively.

Random intercepts were included in the model to account for the correlation between observations within the longitudinal profile of the infection for an individual. More specific, for individual $i$ the (conditional) expected logarithm of the outcome (denoted as $E\left(\mathrm{log}{y}_{ijk}|{z}_{i}\right)$, where $k=\mathrm{1,2},3$ for parasite density, gametocyte density and gametocyte fraction respectively) at time point ${t}_{ij}$ is assumed to be equal to:

2 $$E\left(\mathrm{log}{y}_{ijk}|{z}_{i}\right)={\beta }_{0,k}+{f}_{0,k}\left({t}_{ijk}\right)+{g}_{k}\left({x}_{i,q}\left({t}_{ijk}\right)\right)+{\beta }_{p,k}{x}_{i,p}+{z}_{i}$$

where $$k=1, 2, 3$$ define the outcomes parasite density, gametocyte density and gametocyte fraction respectively, $${f}_{0,k}(.)$$ is the non-linear function which expresses the effect of infection duration and $${g}_{k}(.)$$ is the non-linear function for the effect of the time-varying covariate log parasite density $$\left({x}_{i,q}(t)\right)$$ for outcomes gametocyte density and gametocyte fraction ($$k=2, 3)$$ and 0 otherwise (i.e. $${g}_{1}\left(.\right)=0$$). Regression coefficient $${\beta }_{p,k}$$ is the unknown coefficient for the effect of the $${p}^{th}$$ covariate $${x}_{i,p}$$ for each of the $$P$$ covariates (for individual $$i$$) and $${z}_{i}$$ is the random intercept for the $${i}^{th}$$ individual. Non-linear functions are represented through basis functions and coefficients, e.g. $${f}_{0}\left(s\right)=\sum_{l=1}^{L}{\gamma }_{l}{b}_{l}(s)$$ where $${\gamma }_{l}$$ are unknown regression parameters to be estimated and $${b}_{l}\left(s\right)$$ are chosen basis functions. B-spline basis functions were used.

We first fit models with only the penalized spline for time for each outcome to visualize the baseline expected parasite density, gametocyte density and gametocyte fraction over the duration of infection, i.e. $E\left(\mathrm{log}{y}_{ijk}|{z}_{i}\right)={\beta }_{0,k}+{f}_{0,k}\left({t}_{ijk}\right)+{z}_{i}$. To visualize the (potentially non-linear) effect of parasite density as a time-varying covariate on gametocyte density and gametocyte fraction, we fit a model with a penalized spline for the non-linear effect of log parasite density, i.e. $E\left(\mathrm{log}{y}_{ijk}|{z}_{i}\right)={\beta }_{0,k}+{f}_{0,k}\left({t}_{ijk}\right)+{g}_{k}\left({x}_{i,q}\left({t}_{ijk}\right)\right)+{z}_{i}$, for $k=2, 3$. To assess the effects of the remaining time invariant categorical covariates on each for the outcomes, we fit independent models for each covariate as specified in (2).

We present the results for the time-invariant categorical covariates as density ratios (DRs): $DR=\mathrm{exp}{\beta }_{p,k}$ which quantifies how much larger (multiplicatively) the outcome (parasite and gametocyte densities or gametocyte fraction) is for all levels of the ${p}^{th}$ covariate ${x}_{p}$ relative to its reference category.

## Supplementary Information

Supplementary Information.

## Data Availability

Data from the PRISM2 cohort study is available through a novel open-access clinical epidemiology database resource here: https://clinepidb.org/ce/app/record/dataset/DS_51b40fe2e2. https://clinepidb.org/ce/app/record/dataset/DS_51b40fe2e2 R codes to generate table and figures are available on Github^66^.
