# Supplementary material for: Plasmodium falciparum gametocyte carriage in longitudinally monitored incident infections is associated with duration of infection and human host factors
Source: Sci Rep. 2023 May 1;13:7072. doi: 10.1038/s41598-023-33657-3 (PMC10150352; doi:10.1038/s41598-023-33657-3)
Supplement: Supplementary file 1 — Supplementary Information. [file 41598_2023_33657_MOESM1_ESM.docx]

**Supplemental figures**


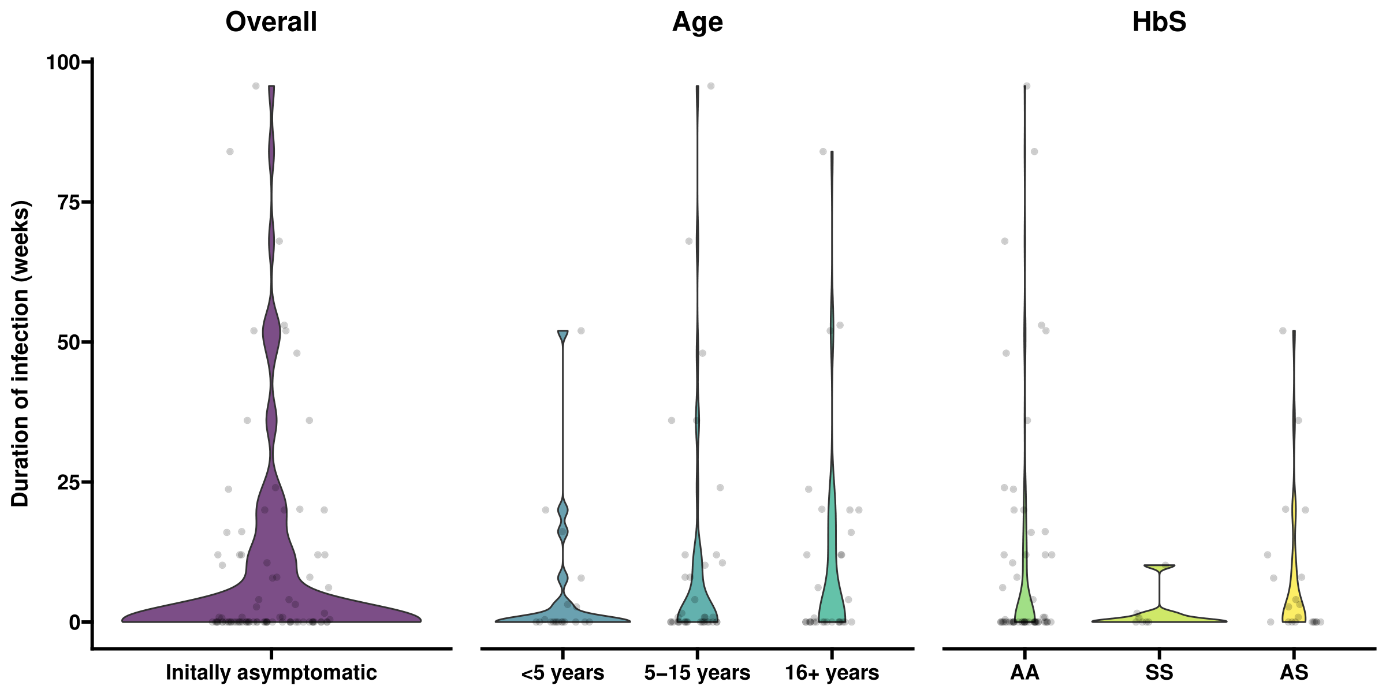


**Figure S1**: **Violin plots of duration of infection in initially asymptomatic (purple), in different age groups <5, 5-15, 16+ years old (blue, petrol, turquoise) and in Hb AA, SS, AS (green, light green, yellow).** There was no association between duration of infection with age (p=0.5049) and Hb (p=0.6648).

**Figure S2: Baseline incidence density/hazard rates for first detection of gametocytes and clearance without gametocyte detection over the time since detected incident malaria infection.** This figure illustrates the unadjusted baseline plots of how the incidence of first detection of gametocytes and also malaria clearance without gametocytes detection evolves over the time since detected infection. The incidence rate is the probability of experiencing either of the events over the time since detection among those who are still event free at that time. The largest incidence of detectable gametocytes occurred at or soon after detection of incident malaria infections (green line) and decreased overtime. Whilst the incidence rate of malaria clearance without detectable gametocytes steadily decreased over the duration of infection.


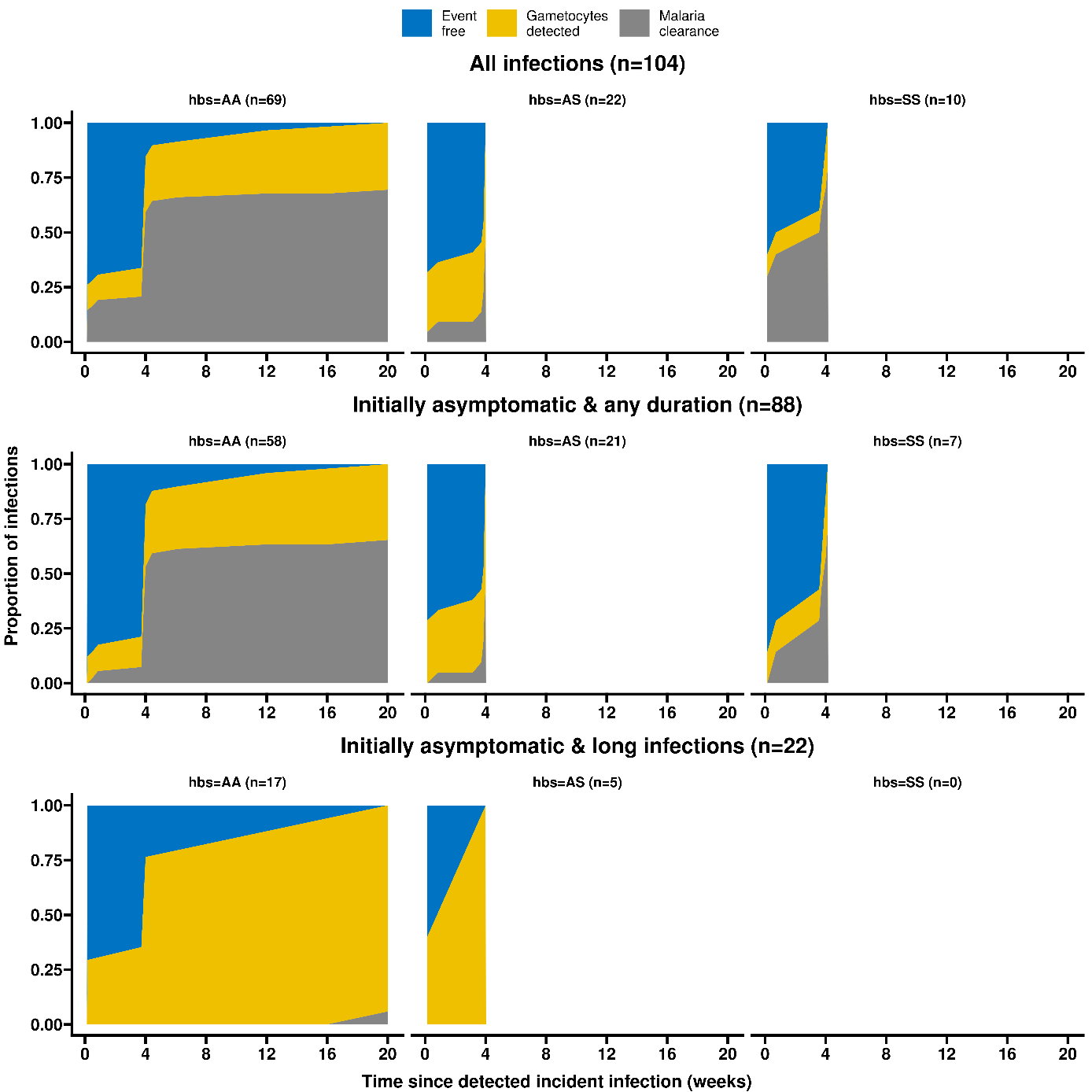


**Figure S3: Gametocytes first detection and malaria clearance without gametocytes detection over time.** This stacked plot shows the cumulative proportion of infections that either had detectable gametocytes (yellow) or cleared their malaria infection without detectable gametocytes over time (grey). Shown in blue is the proportion event free over time. All plots are stratified for HbS where AA (wild type) is shown on the left panel, AS (heterozygote) in the middle panel and SS (homozygote) on the right panel. The top panel is for all 104 infections, of which 3 had missing HbS data, 69 were AA, 22 AS and 10 SS. By 4 weeks approximately half the AS infections had detectable gametocytes and the other half cleared their malaria infection. The middle panel is for the 88 infections that were asymptomatic initially and for all durations of infection, of which 2 HbS data were missing, 58 were AA, 21 AS and 7 SS. 26% (15/57) AA and 52% (11/21) AS infections had detectable gametocytes by 4 weeks. The bottom panel is for the 22 infections that were initially asymptomatic and had >12 weeks total duration of infection, of which 17 were AA, 5 AS and 0 SS. 76% (13/17) AA had detectable gametocytes by 4 weeks and 100% (5/5) AS had detectable gametocytes by 4 weeks.


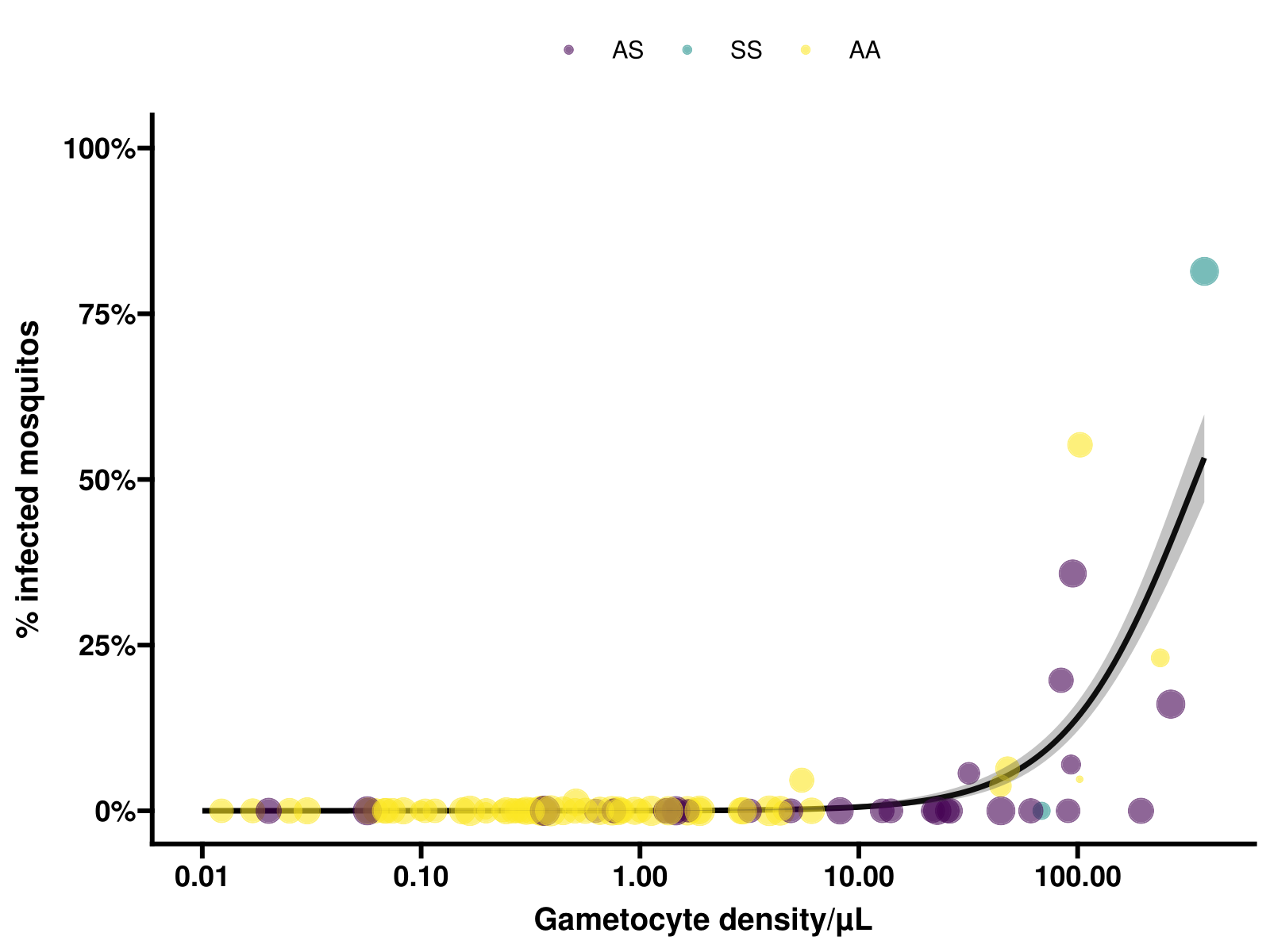


**Figure S4**: **Percentage of infected mosquitoes in relation to gametocyte density expressed per µl for AS (purple), SS (petrol) AA (yellow), individuals**. The size of the dots reflects the number of mosquitoes dissected. From the 21 infectious individuals, 12 had a single feed, 6 had two feeds, 1 had three feeds, 1 had four feeds and 1 had 8 feeds performed. The x-axis shows the gametocyte density (expressed per µl) in log-10 scale.

**Sensitivity analysis-parasite density** **cut off <0.1 parasites per µl**


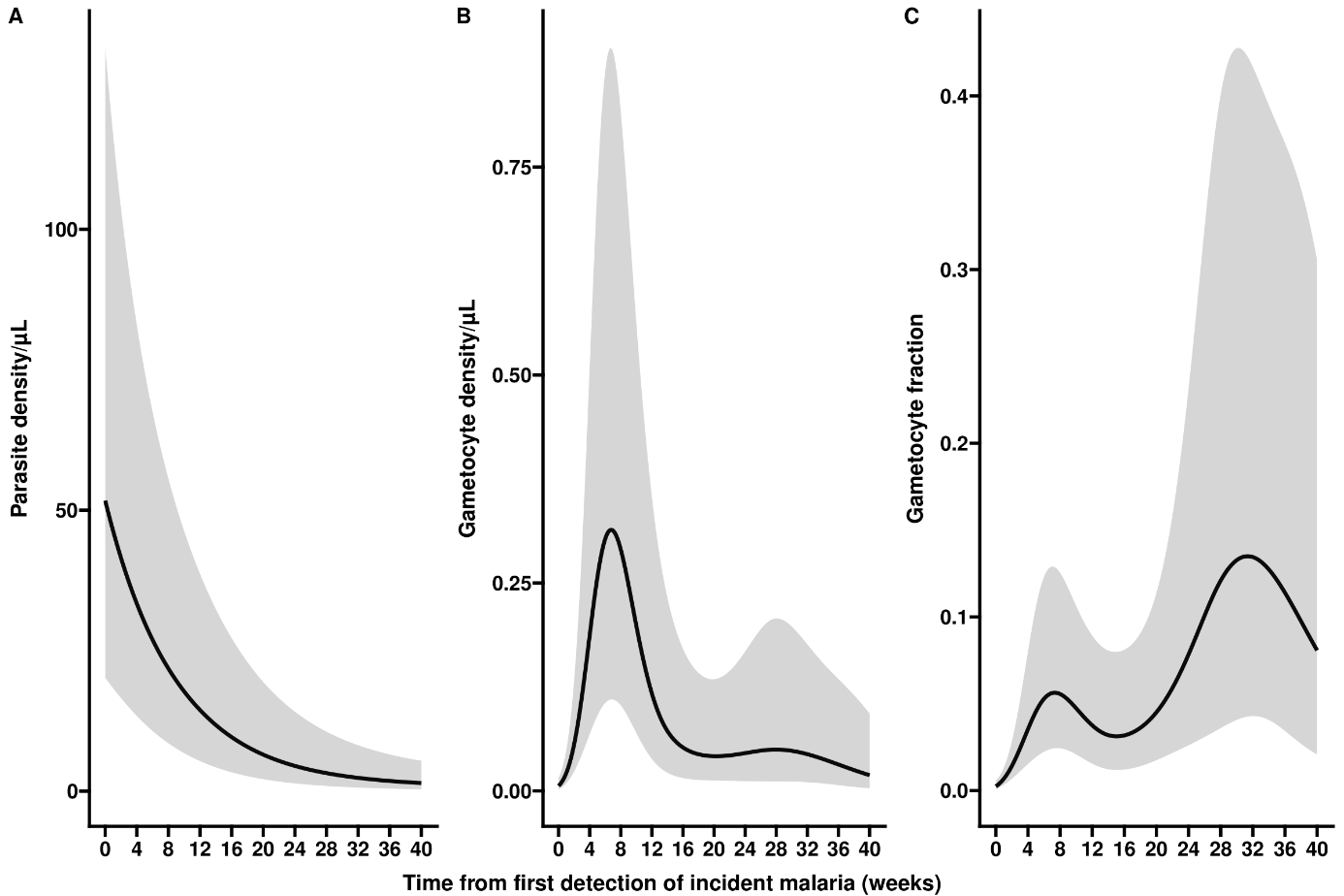


**Figure S5: Parasite density, gametocyte density and gametocyte fraction over the course of incident infections.** Three separate characteristics of infections are presented: parasite density (**A**), gametocyte density (**B**) and gametocyte fraction (**C**). Gametocyte fraction is defined as the proportion of parasites that are gametocytes, estimated as the proportion of the total parasite biomass (i.e. the density estimated by varATS qPCR) that consists of gametocytes (estimated by Ccp4 and PfMGET qRT-PCR). All estimates are presented over time since first detection of incident malaria; all associations are best described by non-linear models.


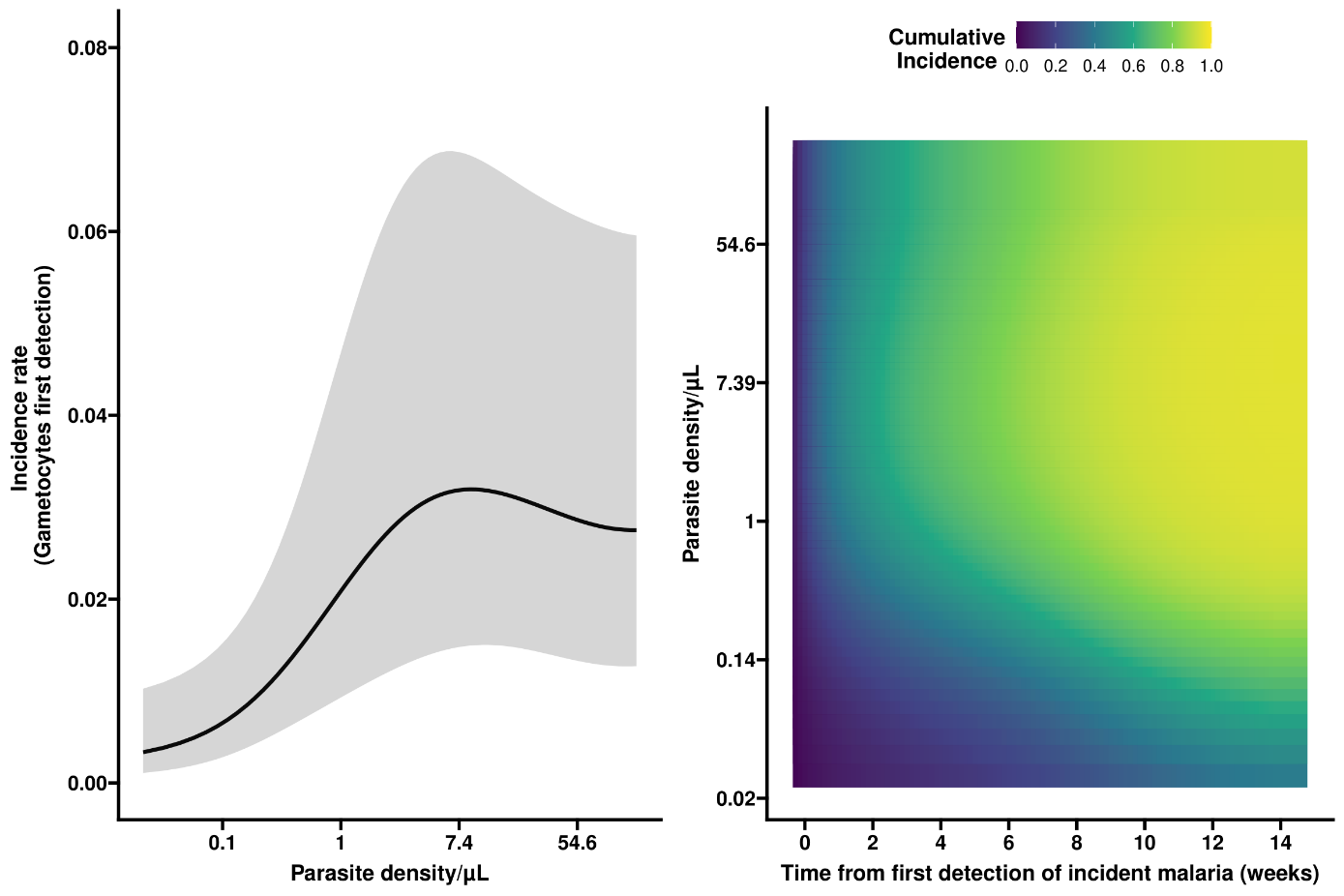


**Figure S6: First detection of gametocytes in relation to parasite density and infection duration.** Panel **A** shows the non-linear association between parasite density/µl (log transformed values) and the incidence of first detection of gametocytes, adjusted for duration of infection. Panel **B** presents the association between parasite density and the duration of incident infection with the cumulative incidence of detectable gametocytes. In Panel **B**, on the y axis the parasite density is expressed per µl, while the x axis describes the time in weeks since the first detection of infection. Low cumulative incidence is shown in blue, whilst cumulative incidences closer to 100% are shown in yellow. Expected cumulative incidences for first detection of gametocytes are presented over the full duration of an infection if parasite densities would be maintained at the level given on the y-axis; the likelihood of having first detection of gametocytes increases with increasing parasite density and longer time since infection

**Sensitivity analysis-parasite density cut off <0.1 parasites per µl**


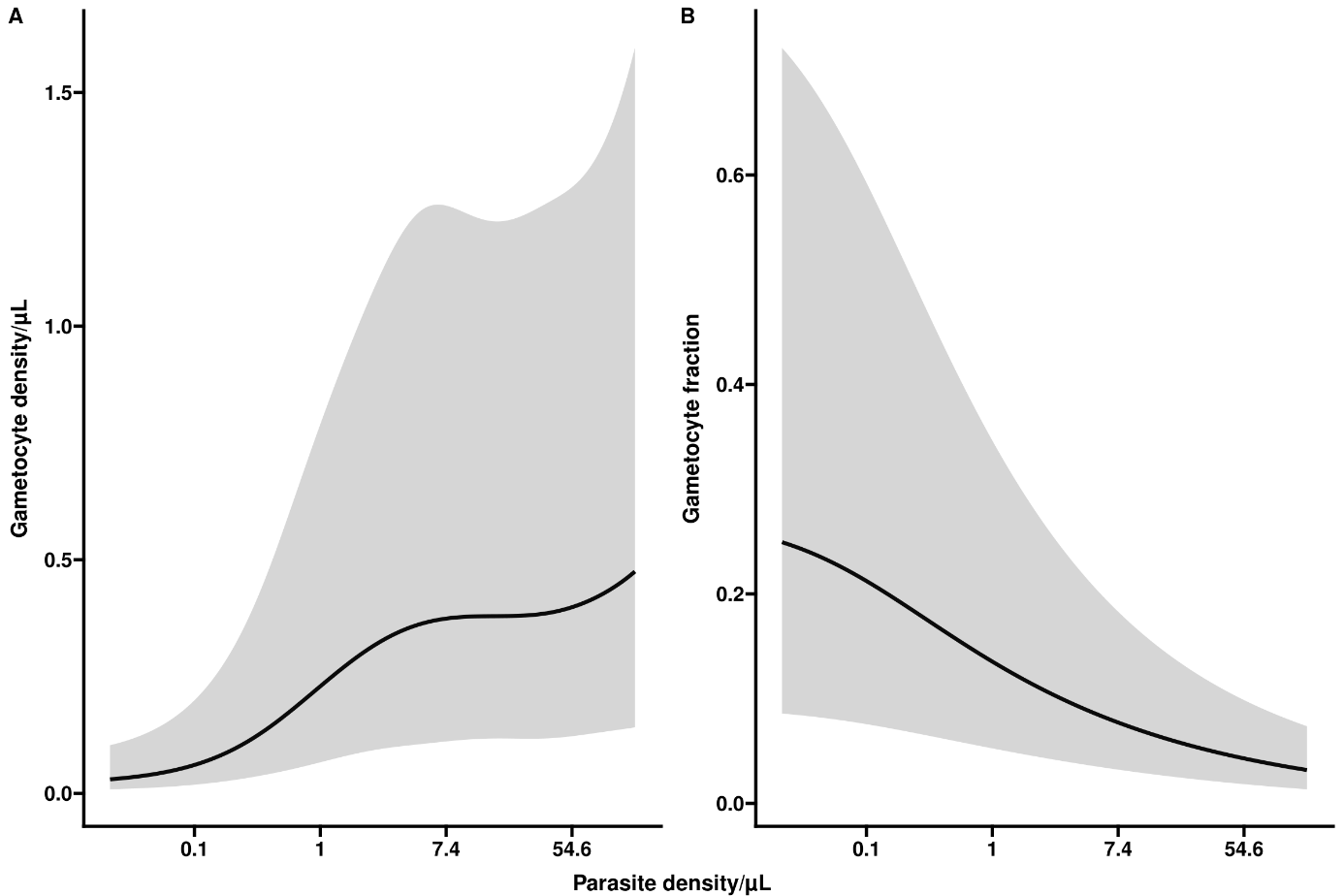


**Figure S7: Gametocyte density and gametocyte fraction in relation to parasite density.** In panel **A**, the association of parasite density with gametocyte density is presented, adjusted for the duration of infection. We observe a positive association between total parasite density and gametocyte density. In panel **B**, the association of parasite density with gametocyte fraction is presented, adjusted for the duration of infection. Higher parasite densities are associated with lower gametocyte fraction at any given moment in time. Visits when the infection was detected without detectable gametocytes were included in these analyses.

**Sensitivity analysis for all infections that remained asymptomatic**


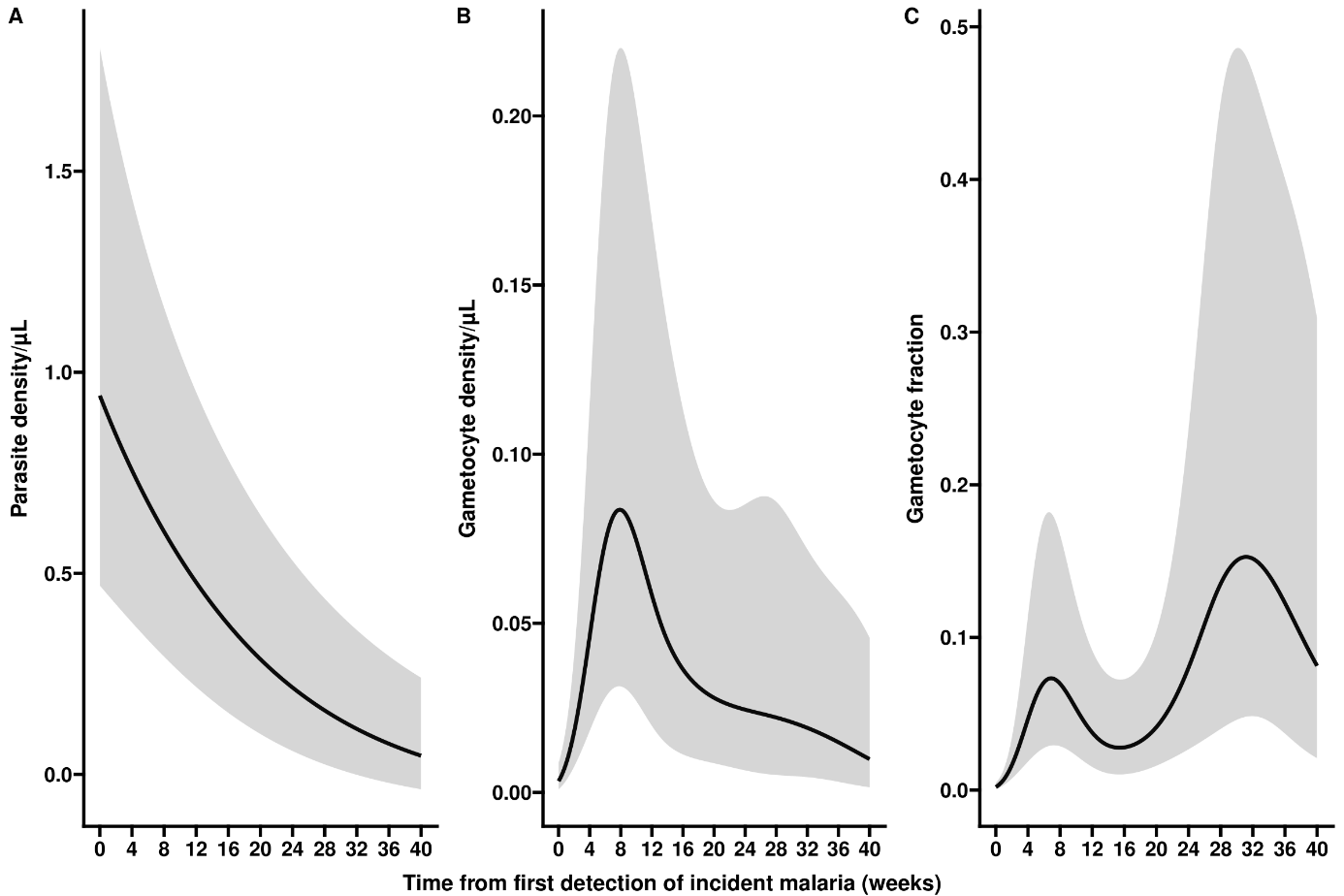


**Figure S8: Parasite density, gametocyte density and gametocyte fraction over the course of incident infections.** Three separate characteristics of infections are presented: parasite density (**A**), gametocyte density (**B**) and gametocyte fraction (**C**). Gametocyte fraction is defined as the proportion of parasites that are gametocytes, estimated as the proportion of the total parasite biomass (i.e. the density estimated by varATS qPCR) that consists of gametocytes (estimated by Ccp4 and PfMGET qRT-PCR). All estimates are presented over time since first detection of incident malaria; all associations are best described by non-linear models.


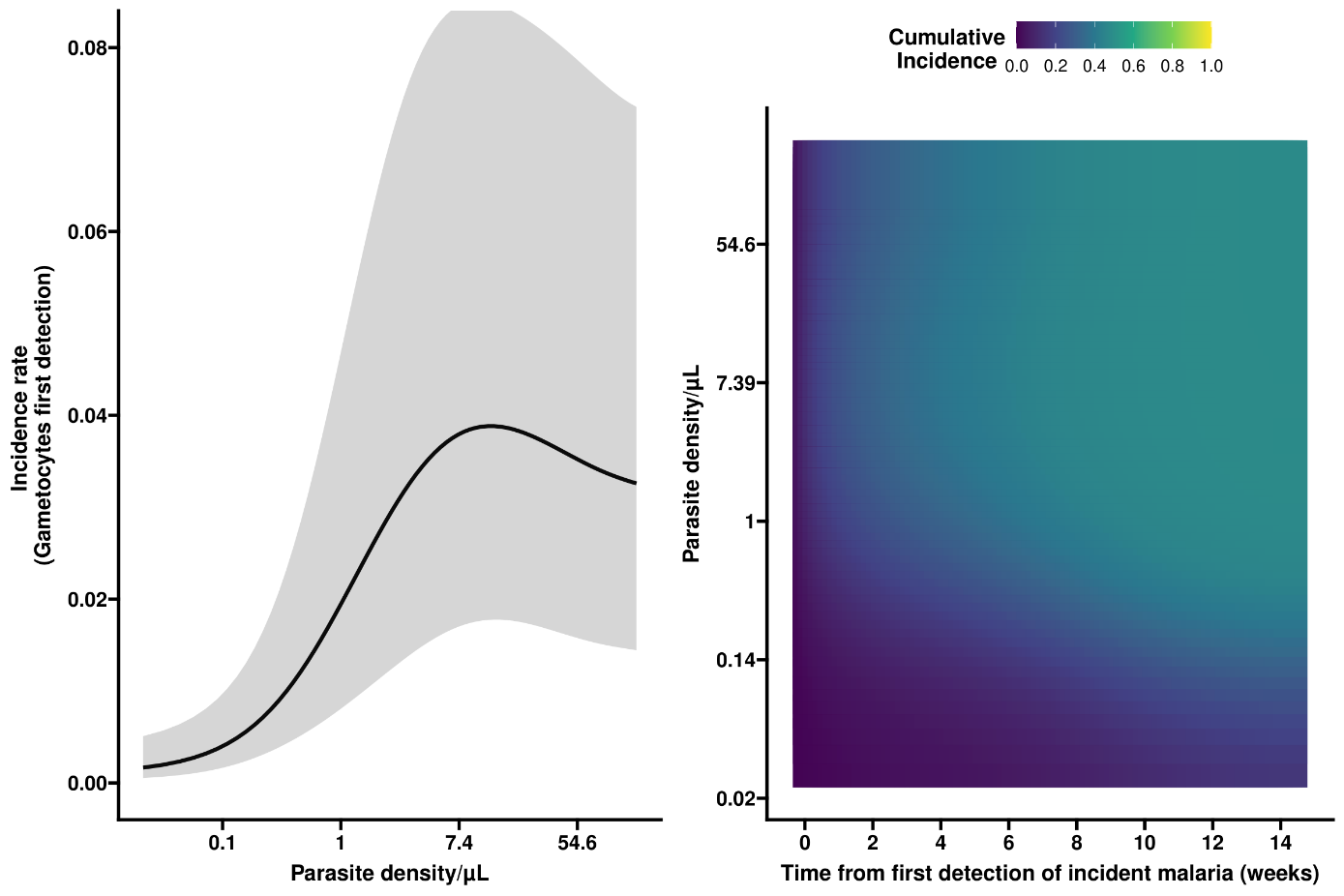


**B**

B

**A**

Figure S9 First detection of gametocytes in relation to parasite density and infection duration. Panel A shows the non-linear association between parasite density/µl (natural log transformed values) and the incidence of first detection of gametocytes, adjusted for duration of infection. Panel B presents the association between parasite density and the duration of incident infection with the cumulative incidence of detectable gametocytes. In panel B, on the y axis the parasite density is expressed per µl, while the x axis describes the time in weeks since the first detection of infection. Low cumulative incidence is shown in blue, whilst cumulative incidences closer to 100% are shown in yellow. Expected cumulative incidences for first detection of gametocytes are presented over the full duration of an infection if parasite densities would be maintained at the level given on the y-axis; the likelihood of having detectable gametocytes increases with increasing parasite density and longer time since infection.


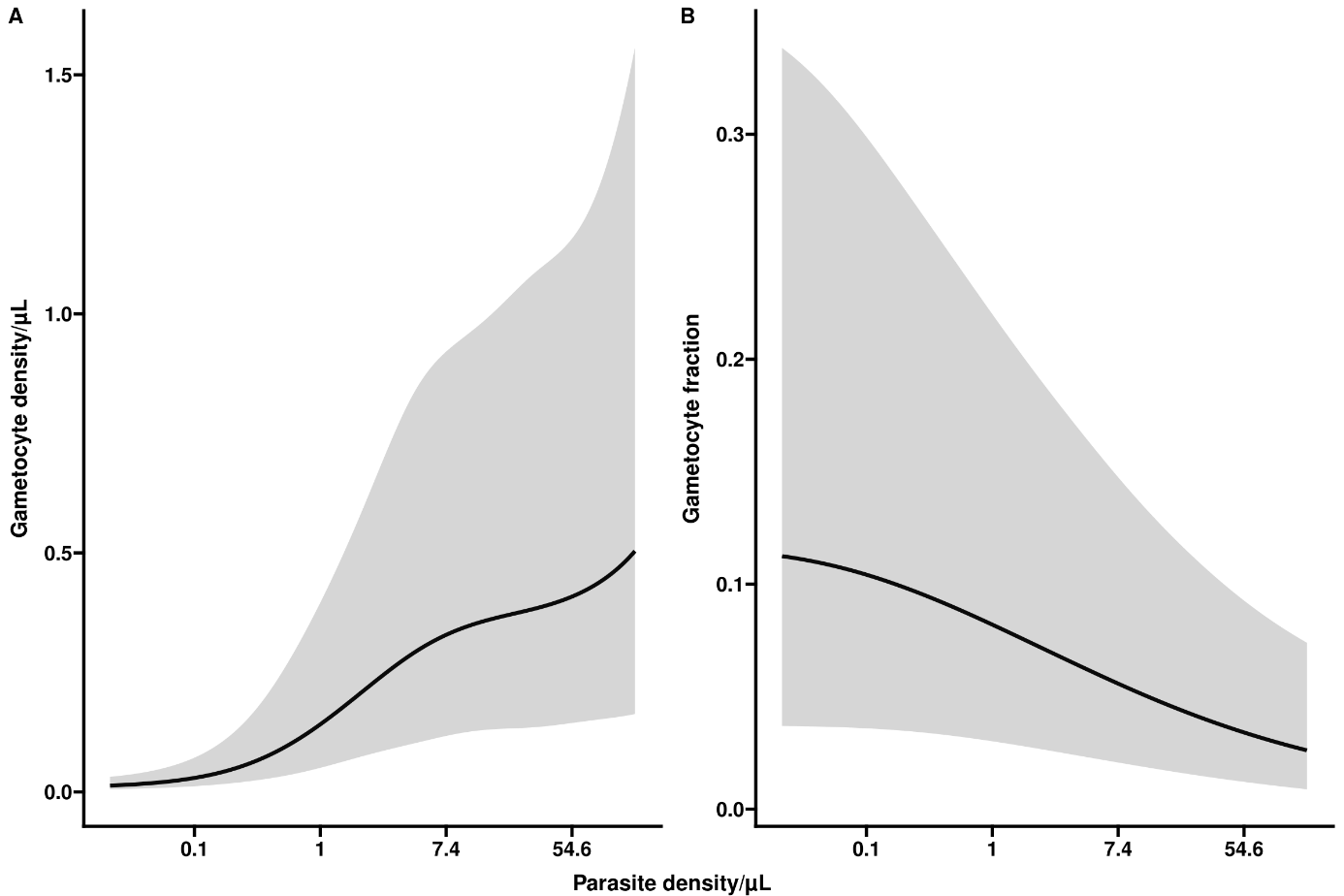


**Figure S10: Gametocyte density and gametocyte fraction in relation to parasite density.** In panel **A**, the association of parasite density with gametocyte density is presented, adjusted for the duration of infection. We observe a positive association between total parasite density and gametocyte density. In panel **B**, the association of parasite density with gametocyte fraction is presented, adjusted for the duration of infection. Higher parasite densities are associated with lower gametocyte fraction at any given moment in time. Visits when the infection was detected without detectable gametocytes were included in these analyses.

**
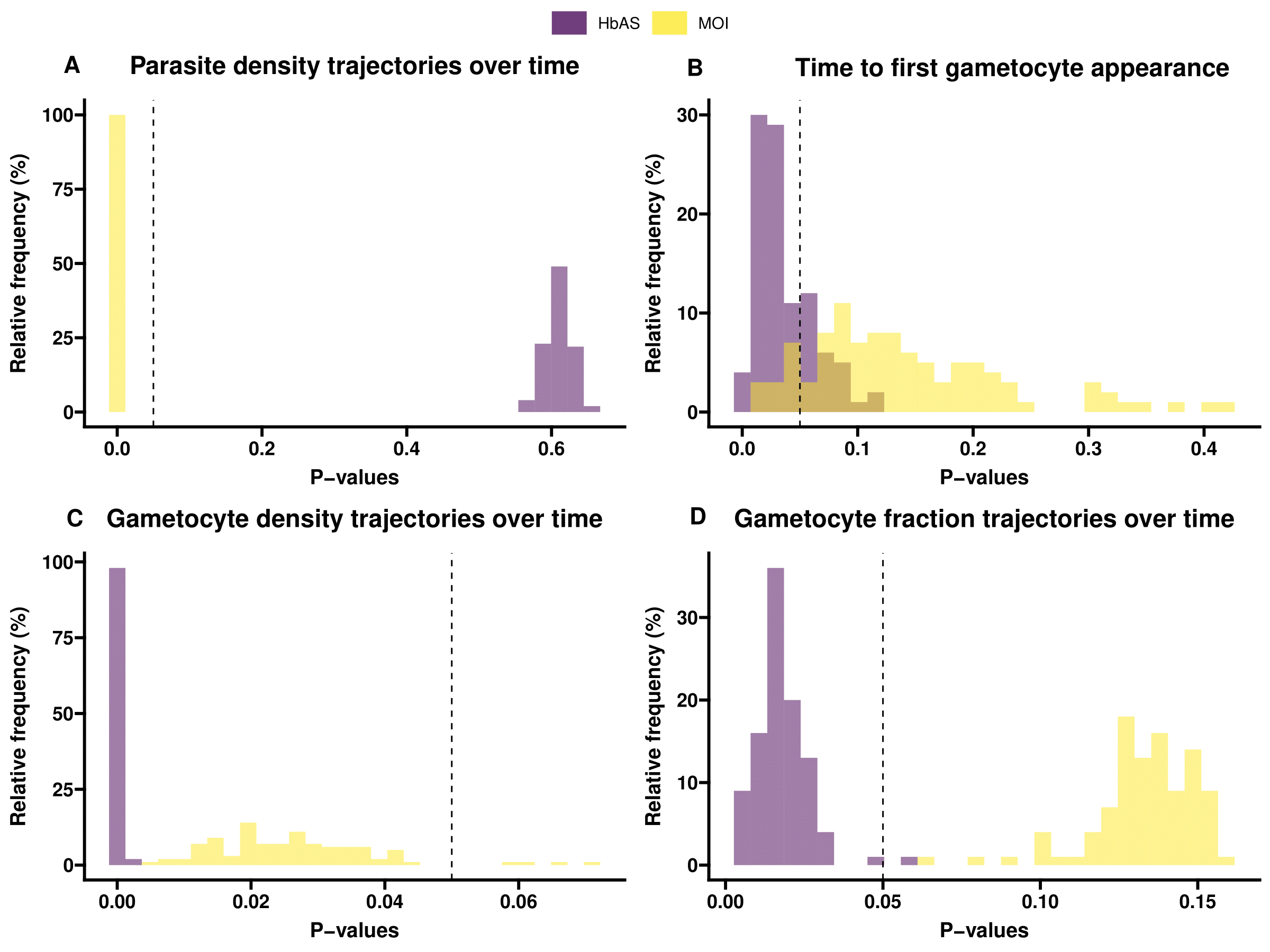
**

**Figure S11: Distribution of p-values from one hundred imputed datasets to evaluate the sensitivity to HbAS and multiplicity of infection (MOI>1) to interval-censoring.** All underlying exact times, time of incident malaria, time of first gametocyte appearance and time of recovery are interval-censored. Ignoring interval-censoring may lead to underestimated standard errors and thus inflated type I error rates. Exact times for each analyses were imputed 100 times from a uniform distribution within the interval, and then each imputed dataset was analyzed for each outcome (**A**) parasite densities over time, (**B**) time to first gametocyte appearance, (**C**) gametocyte densities over time and (**D**) gametocyte fraction over time and the p-values for HbAS (purple) and MOI>1 (yellow) were reported. Dashed vertical lines indicated the 5% level of significance cutoff.

**
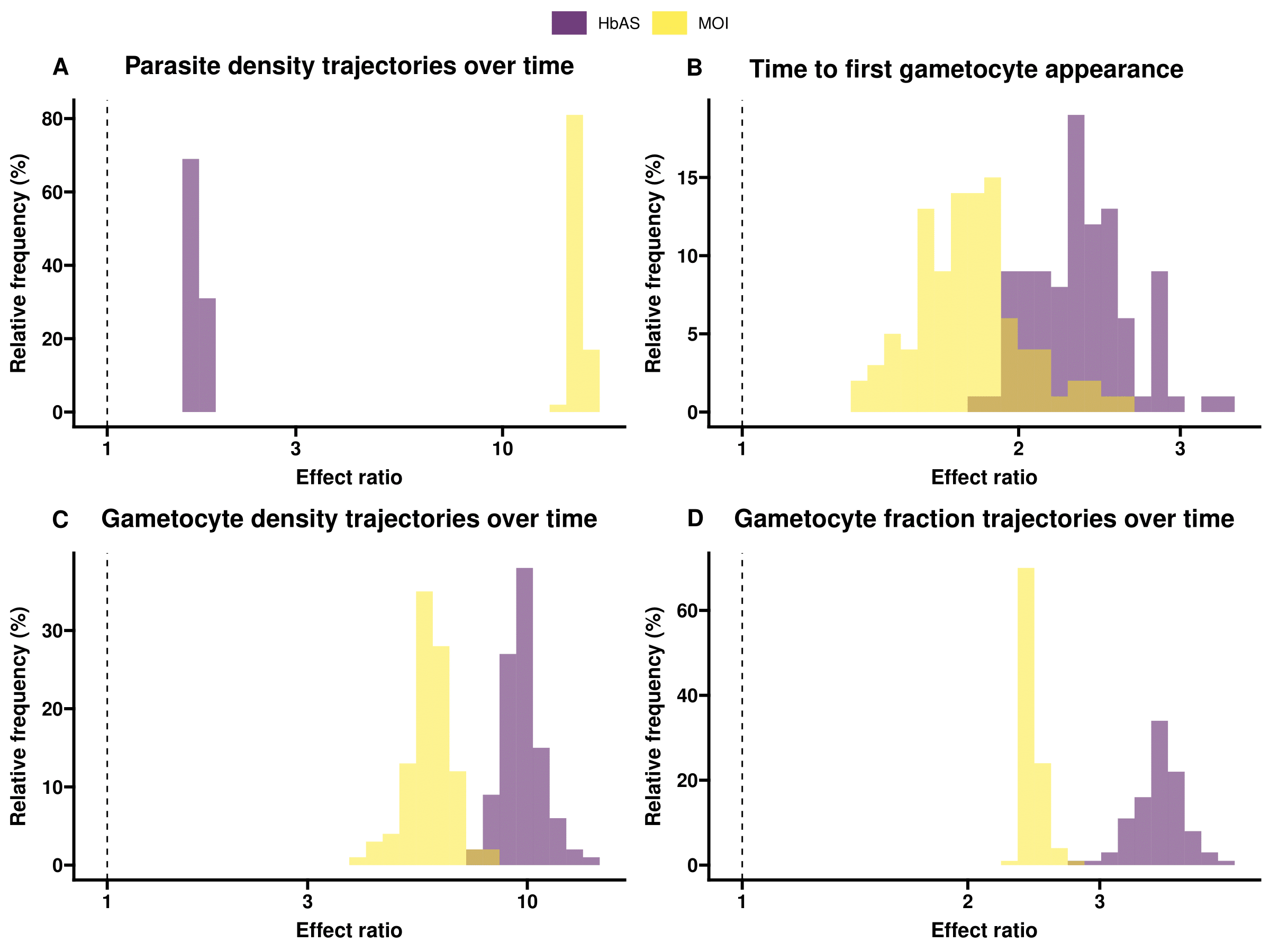
**

**Figure S12: Distribution of effects (density ratios or hazard ratios) from one hundred imputed datasets to evaluate the sensitivity to HbAS and MOI>1 to interval-censoring.** All underlying exact times, time of incident malaria, time of first gametocyte appearance and time of recovery are interval-censored. Ignoring interval-censoring may lead to underestimated standard errors and thus inflated type I error rates. Exact times for each analyses were imputed 100 times from a uniform distribution within the interval, and then each imputed dataset was analyzed for each outcome (**A**) Parasite densities over time, (**B**) time to first gametocyte appearance, (**C**) gametocyte densities over time and (**D**) gametocyte fraction over time) and the effects (density ratios or hazard ratios) for HbAS (purple) and MOI>1 (yellow) were reported. Dashed vertical lines at effect of 1 indicate no difference in the hazards/densities compared with the corresponding reference groups (HbAA or MOI=1).

|  | **Parasite density** | | **Incidence of detectable gametocytes** | | **Gametocyte**  **density** | | **Gametocyte**  **fraction** | | **Malaria**  **clearance (without detectable gametocytes)** | |
| --- | --- | --- | --- | --- | --- | --- | --- | --- | --- | --- |
|  | **DR (95% CI)** | **p-value** | **HR (95% CI)** | **p-value** | **DR (95% CI)** | **p-value** | **DR (95% CI)** | **p-value** | **HR (95% CI)** | **p-value** |
| **Age (<5 years=reference group)** | | | | | | | | | | |
| **5-15 years** | 0.96 (0.2, 4.73) | 0.9601 | 2.13 (0.65, 7) | 0.2054 | 1.44 (0.34, 5.98) | 0.6121 | 0.97 (0.22, 4.25) | 0.9725 | 1.18 (0.55, 2.53) | 0.6736 |
| **16+ years** | 1 (0.2, 4.95) | 0.9963 | 2.02 (0.56, 7.31) | 0.2727 | 1.03 (0.25, 4.34) | 0.9625 | 1.42 (0.32, 6.22) | 0.6359 | 0.8 (0.35, 1.82) | 0.5834 |
| **Gender (female=reference group)** | | | | | | | | | | |
| **Male** | 1.24 (0.38, 4.06) | 0.7151 | 1.25 (0.51, 3.07) | 0.6172 | 1.71 (0.61, 4.82) | 0.3 | 1.43 (0.5, 4.11) | 0.5013 | 0.98 (0.51, 1.86) | 0.9486 |
| **HB genotype (AA =reference group)** | | | | | | | | | | |
| **SS** | 0.13 (0.01, 2.06) | 0.1409 | 0 (0, Inf) | 1 | 1.86 (0.1, 35) | 0.671 | 0.26 (0.01, 5.22) | 0.3695 | 0.89 (0.22, 3.56) | 0.8701 |
| **AS** | 3.13 (0.81, 12.06) | 0.091 | 3.02 (1.18, 7.74) | 0.0188 | 8.45 (2.77, 25.82) | 0.0001 | 4.31 (1.29, 14.37) | 0.0152 | 1.16 (0.5, 2.67) | 0.7224 |
| **Clonality (monoclonal infection=reference group)** | | | | | | | | | | |
| **Polyclonal** | 11.47 (2.8, 47.02) | 0.0005 | 2.75 (0.77, 9.74) | 0.1103 | 4.46 (0.83, 24.06) | 0.0759 | 3.07 (0.54, 17.38) | 0.195 | 1.03 (0.15, 7.06) | 0.9729 |

**Table S1. Sensitivity analysis of asymptomatic infections.** In these analyses all infections that were either symptomatic at the moment of infection detection or at any time-point during follow-up were removed from the analyses. This reduced the study population from 104 monitored infections to 76 infections.

|  | **Parasite density** | | **Incidence of detectable gametocytes** | | **Gametocyte**  **density** | | **Gametocyte**  **fraction** | | **Malaria**  **clearance (without detectable gametocytes)** | |
| --- | --- | --- | --- | --- | --- | --- | --- | --- | --- | --- |
|  | **DR (95% CI)** | **p-value** | **HR (95% CI)** | **p-value** | **DR (95% CI)** | **p-value** | **DR (95% CI)** | **p-value** | **HR (95% CI)** | **p-value** |
| **Age (<5 years=reference group)** | | | | | | | | | | |
| **5-15 years** | 1.06 (0.11, 10.08) | 0.959 | 1.89 (0.7, 5.1) | 0.1982 | 1.49 (0.27, 8.3) | 0.6423 | 1.19 (0.31, 4.51) | 0.7963 | 0.68 (0.28, 1.63) | 0.375 |
| **16+ years** | 0.14 (0.01, 1.52) | 0.0983 | 1.22 (0.4, 3.74) | 0.7165 | 0.69 (0.11, 4.39) | 0.6879 | 1.06 (0.25, 4.47) | 0.9337 | 0.17 (0.06, 0.47) | 0.0005 |
| **Gender (female=reference group)** | | | | | | | | | | |
| **Male** | 0.46 (0.07, 2.94) | 0.403 | 0.95 (0.44, 2.05) | 0.8855 | 1.18 (0.31, 4.54) | 0.8086 | 1.24 (0.45, 3.42) | 0.6742 | 0.62 (0.27, 1.42) | 0.2505 |
| **HB genotype (AA =reference group)** | | | | | | | | | | |
| **SS** | 106.93 (3.52, 3247.04) | 0.0062 | 1.09 (0.22, 5.38) | 0.9117 | 1.34 (0.11, 16.55) | 0.8137 | 3.55 (0.46, 27.64) | 0.2175 | 1.38 (0.4, 4.85) | 0.6038 |
| **AS** | 1.26 (0.16, 10.18) | 0.8227 | 2.69 (1.14, 6.33) | 0.0209 | 12.55 (2.94, 53.47) | 0.0005 | 3.86 (1.2, 12.36) | 0.0204 | 0.28 (0.09, 0.92) | 0.0326 |
| **Clonality (monoclonal infection=reference group)** | | | | | | | | | | |
| **Polyclonal** | 13.62 (2.34, 79.24) | 0.003 | 2.93 (1.15, 7.42) | 0.021 | 5.97 (1.39, 25.6) | 0.014 | 2.37 (0.78, 7.22) | 0.1218 | 0.42 (0.18, 0.97) | 0.0391 |

**Table S2. Sensitivity analysis excluding infections with low parasite densities.** For these analyses, all infections with an initial parasite density below 0.1 parasites per µl were excluded, reducing the study population from 104 monitored infections to 73 infections.

|  | **Incident initially symptomatic infections** | **Incident initially asymptomatic infections** | **p-value** |
| --- | --- | --- | --- |
| **At moment of infection detection** | | | |
| **Proportion of infections in each class** | 21.9% (16/73) | 78.1% (57/73) |  |
| **Hb genotype**  **AA**  **SS**  **AS** | 1 missing  73.3% (11/15)  20% (3/15)  6.7% (1/15) | 2 missing  65% (37/57)  5.2% (3/57)  28% (16/57) | p=0.0548 |
| **Geometric mean parasite density; parasites/µL (95% CI)** | 19817.75 (3099.15, 126726.26) | 6.98 (2.61-18.65) | p<0.0001 |
| **Gametocyte prevalence** | 6.2% (1/16) | 24.6% (14/57) | p=0.1650 |
| **During infection** | | | |
| **Geometric mean peak parasite density; parasites/µL (95% CI)** | 19817.75 (3171.58, 123832.07) | 121.19 (45.90, 319.95) | p<0.0001 |
| **Polyclonal infection *** | 50% (8/16) | 34% (17/50)  7 missing*** | p=0.2505 |
| **Number of clones, median (range)**** | 1.5 (1 – 5) | 1 (1 – 5) | p=0.1274 |
| **Percentage receiving treatment** | 100% (16/16) | 21.1% (12/57) | p<0.0001 |

**Table S3. Characteristics of incident infections that presented with symptoms and initially asymptomatic (cut off <0.1 parasites per µl****).**

*Polyclonal infection was defined as having at least 2 parasite clones based on AMA-1 amplicon sequencing.

**Number of clones was defined as the total number of clones detected by AMA-1 amplicon sequencing. Polyclonal infection and the number of parasite clones were measured across the entire duration of infection; for symptomatic incident infections this equaled the first time-point of detection since treatment was immediately provided.

***For asymptomatic incident infections, AMA-1 sequencing was unsuccessful for 37 infections.

| **At moment of infection detection** | **All infections** |
| --- | --- |
|  | N=76 |
| **Hb genotype**  **AA**  **SS**  **AS** | 1 missing  69.7% (53/76)  5.3% (4/76)  23.7% (18/76) |
| **Geometric mean parasite density; parasites/µL (95% CI)** | 0.41 (0.17, 1.02) |
| **Gametocyte prevalence** | 14.4% (11/76) |
| **During infection** | |
| **Geometric mean peak parasite density; parasites/µL (95% CI)** | 1.65 (0.57, 4.76) |
| **Polyclonal infection*** | 30.8% (12/39)  37 missing*** |
| **Number of clones, median (range)**** | 1 (1 – 5) |
| **Percentage receiving treatment** | 0% (0/76) |

**Table S4. Characteristics of asymptomatic incident infections** (symptomatic infections at the moment of infection detection or at any time-point during follow-up were removed from the analyses)**.**

*Polyclonal infection was defined as having at least 2 parasite clones based on AMA-1 amplicon sequencing.

**Number of clones was defined as the total number of clones detected by AMA-1 amplicon sequencing. Polyclonal infection and the number of parasite clones were measured across the entire duration of infection; for symptomatic incident infections this equaled the first time-point of detection since treatment was immediately provided.

***For asymptomatic incident infections, AMA-1 sequencing was unsuccessful for 37 infections.
